# Harnessing Bone-Liver Crosstalk: A Dual-Action LYTAC Approach for Bone-Specific Accumulation and Liver-Specific Protein Degradation in Bone Disorders

**DOI:** 10.1021/jacsau.5c00827

**Published:** 2025-11-20

**Authors:** Yuan Ma, Gubu Amu, Yufei Pan, Hewen Jiang, Sifan Yu, Huarui Zhang, Zefeng Chen, Hang Luo, Chuanxin Zhong, Xin Yang, Xiaohui Tao, Yihao Zhang, Yuanyuan Yu, Aiping Lu, Luyao Wang, Baoting Zhang, Ge Zhang

**Affiliations:** † School of Chinese Medicine, Faculty of Medicine, The Chinese University of Hong Kong, New Territories, Hong Kong SAR 999077, China; ‡ Law Sau Fai Institute for Advancing Translational Medicine in Bone & Joint Diseases, School of Chinese Medicine, 26679 Hong Kong Baptist University, Kowloon City, Hong Kong SAR 999077, China; § Aptacure Therapeutics Limited, New Territories, Hong Kong SAR 999077, China

**Keywords:** Aptamer, N-Acetylgalactosamine, Sclerostin, Osteogenesis imperfecta, Lysosomal targeting chimera

## Abstract

Despite significant progress in extracellular targeted protein degradation (eTPD), existing approaches rarely achieved tissue-specific drug accumulation while maintaining efficient systemic clearance, a critical challenge in treating bone disorders. In this study, we introduced GalNAc-Apc001, a novel aptamer-based lysosome-targeting chimera (LYTAC) that uniquely combined bone-specific retention with hepatocyte-mediated clearance through a spatiotemporally controlled mechanism. By conjugating a tri-N-acetylgalactosamine (GalNAc) moiety to a bone-homing sclerostin aptamer (Apc001), we engineered a bifunctional molecule capable of accumulating in bone via hydroxyapatite binding, capturing circulating sclerostin with high affinity and directing it to hepatocytes for ASGPR-mediated lysosomal degradation. In the absence of ASGPR-positive cells, GalNAc-Apc001 functioned via the conventional aptamer mechanism of binding inhibition, demonstrating efficacy comparable to that of Apc001 but notably lower than that of a sclerostin antibody. However, in ASGPR-positive cell coculture systems, GalNAc-Apc001 achieved a 40% greater activation of the Wnt signaling pathway compared to the sclerostin antibody, effectively reversing sclerostin-mediated inhibition (96 vs 60% recovery). Pharmacologically, GalNAc-Apc001 exhibited superior therapeutic efficacy by mitigating the suppressive effects of sclerostin on Wnt signaling, upregulating bone formation markers, and enhancing bone mass in a *Col1a2* ^*+/G610C*^ osteogenesis imperfecta mouse model. These findings provided compelling mechanistic evidence that the spatiotemporal control of protein degradation could resolve the inherent trade-off between tissue targeting and systemic clearance, supporting the clinical potential of GalNAc-Apc001 in bone disorders.

## Graphical abstract

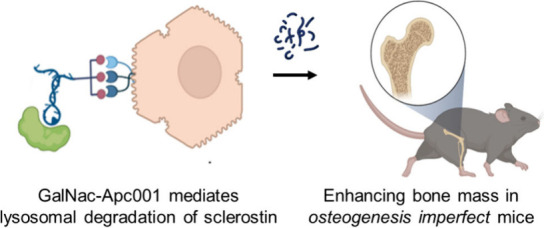

## Introduction

Therapeutic aptamers are increasingly recognized as a novel class of molecular entities with the potential to surpass therapeutic antibodies, owing to their ease of discovery, straightforward synthesis, and low immunogenicity.^1^ Despite these advantages, their limited in vivo efficacy has impeded progress in the field, with only two aptamer-based drugs, pegaptanib and Izervay, approved by the U.S. Food and Drug Administration (FDA) to date.^2^ Aptamers generated through the Systematic Evolution of Ligands by Exponential Enrichment (SELEX) process are primarily selected for their binding affinity to target molecules^3^ rather than their therapeutic potency. Consequently, many SELEX-derived aptamers exhibit minimal or no inhibitory effects *in vivo*. In our previous work, we developed several strategies to enhance aptamer efficacy, including dihydrogen bond delivery systems,^4–6^ noncovalent hydrophobic modification technologies^7–9^ and covalent cross-linking technologies.^10^ However, these approaches have poor batch stability and are time-consuming and labor-intensive.^11^ It is desirable to develop a simple and generalizable approach to significantly enhance the *in vivo* therapeutic efficacy of aptamers.

The clinical application of aptamers faces additional challenges, including poor serum stability and rapid renal clearance.^12,13^ Unmodified aptamers often have extremely short half-lives, with some degrading within minutes in circulation.^14^ These limitations can be mitigated through strategic chemical modifications, as demonstrated by pegaptanib and Izervay, which incorporate 2′-fluoro or 2′-methoxy substitutions and 3′-inverted deoxythymidine caps to confer resistance to nuclease degradation.^15^ Moreover, aptamers typically have a molecular weight (∼20 kDa) below the renal filtration threshold (30–50 kDa), leading to rapid clearance from the bloodstream.^16^ To address these pharmacokinetic limitations, tissue-specific accumulation strategies have been explored. While albumin conjugation can extend the circulation time to several days, it often results in nonspecific tissue distribution.^17^ In contrast, bone-targeting approaches exploit the affinity of aptamers for hydroxyapatite, the mineral matrix of bone, enabling high-specificity binding and prolonged retention.^18^ Our previous work on sclerostin-targeting aptamer Apc001 (aptscl56) applied these principles, incorporating 2′-fluoro/2′-methoxy and 3′-inverted deoxythymidine modifications to enhance stability and achieve efficient bone-specific accumulation.^18,19^ This strategy effectively addressed the dual challenges of serum instability and renal clearance that have historically limited aptamer-based therapeutics.

Aptamers exert their inhibitory effects primarily through an occupancy-driven mechanism, which inherently limits their maximal efficacy. This constraint may reduce their therapeutic utility in diseases where nearly complete target suppression is clinically required.^20^ In recent years, targeted protein degradation (TPD) has emerged as a promising therapeutic modality due to its superior inhibitory efficiency compared to that of traditional occupancy-based inhibitors.^21^ While intracellular TPD (iTPD) technologies such as PROTACs and molecular glues have been extensively studied,^22^ research on extracellular targeted protein degradation (eTPD) remains in its early stage.^23^ eTPD technologies typically function by trafficking proteins of interest (POIs) to lysosomes for degradation via bifunctional molecules that induce the proximity between POIs and lysosome-targeting receptors (LTRs). To date, various LTRs have been exploited for eTPD, including the Fc receptor,^24^ cation-independent mannose-6-phosphate receptor (CI-M6PR),^25^ asialoglycoprotein receptors (ASGPR),^26^ transmembrane E3 ligases,^27^ cytokine receptors,^28^ integrin,^29^ scavenger receptors,^30^ a transferrin receptor,^31^ and glucose transporter Glut1.^32^

ASGPR stands out for its exclusive high expression in liver cells and its primary role in degrading aging proteins in circulation. As such, ASGPR-based eTPD is considered to be one of the most promising approaches for therapeutic development, offering broad applicability and favorable safety.^33^ Moreover, insights from siRNA and antisense oligonucleotide (ASO) drug development suggest that ASGPR-targeted delivery of oligonucleotides can significantly reduce renal clearance and off-target tissue distribution, thereby enhancing the in vivo efficacy of oligonucleotide-based drugs.^34^ Based on these findings, we hypothesized that conjugating aptamers with ASGPR-targeting N-acetylgalactosamine (GalNAc) to construct aptamer-based eTPD molecules would be a straightforward and effective strategy to simultaneously optimize both *in vivo* pharmacodynamic and pharmacokinetic properties. In 2023, Wu et al. successfully established the proof of concept for aptamer-based LYTACs (Apt-LYTACs) for *in vitro*-targeted protein degradation in cancerous cell lines.^35^ However, the therapeutic efficacy of aptamer-based LYTACs *in vivo* and their applicability to metabolic bone disorders remained unexplored. Crucially, the fundamental challenge of achieving tissue-specific drug accumulation while maintaining efficient systemic clearance of target protein remained challenging.

In this study, we utilized sclerostin-targeting aptamer Apc001 to test our hypothesis. Sclerostin is a canonical antagonist of the Wnt signaling pathway and is a key negative regulator of bone formation. Its inhibition represents a promising therapeutic strategy for bone-related diseases such as osteoporosis, osteogenesis imperfecta (OI), and hypophosphatasia.^36,37^ Our group previously identified Apc001 as a potential sclerostin inhibitor, but its occupancy-driven mechanism yielded only moderate *in vivo* efficacy, which was lower than that of the equivalent sclerostin antibody (romosozumab).^19,38^ To overcome these limitations, we utilized a dual-targeting strategy by engineering GalNAc-Apc001 (Figure 1), a novel LYTAC that uniquely combined bone-specific homing via hydroxyapatite binding with hepatocyte-mediated degradation of sclerostin. This spatiotemporally controlled mechanism enabled potent drug retention at the pathological site (bone) while leveraging the liver for efficient clearance, which we rigorously validated by demonstrating superior therapeutic efficacy over both the parent aptamer and a sclerostin antibody in an osteogenesis imperfecta mouse model. Our work advanced the Apt-LYTAC platform from a general degradation tool into a targeted therapeutic strategy that resolved the inherent trade-off between tissue targeting and systemic clearance for the treatment of bone disorders.

**Figure 1:**
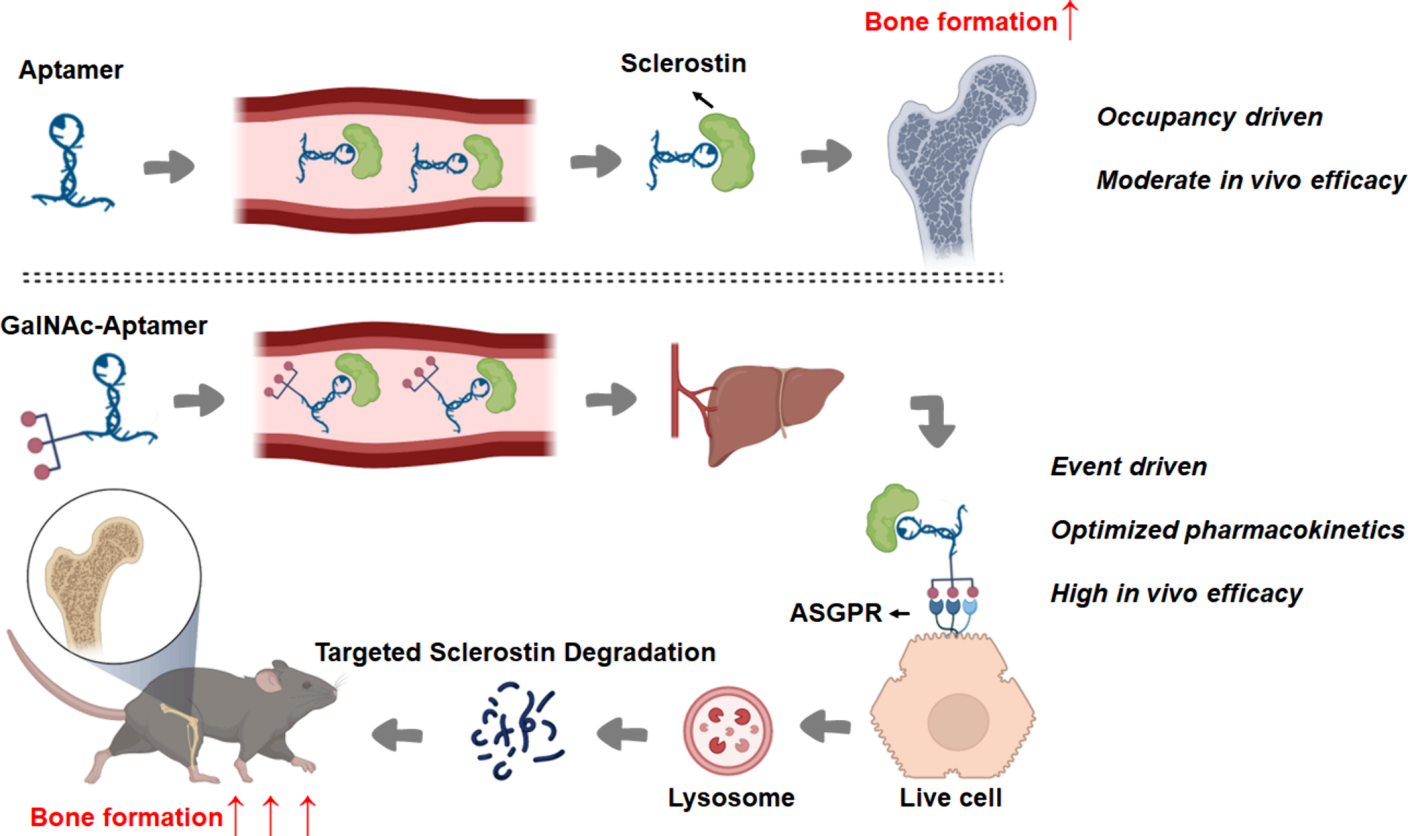
Schematic diagram comparing the difference in the mechanism of action between therapeutic aptamers and aptamer-based eTPD molecules. The sclerostin aptamer can decrease sclerostin activity by an occupancy-driven mechanism, resulting in a moderate promotion of bone formation. GalNAc-aptamer, an aptamer-based eTPD molecule, can completely abolish sclerostin activity by degrading sclerostin with an event-driven mechanism, resulting in a significant promotion of bone formation.

## Results and Discussion

### Construction and Hepatocyte-Specific Internalization of GalNAc-Apc001 Conjugates

GalNAc-DNA conjugates were synthesized as outlined in Scheme S1. Briefly, a tri-GalNAc succinimidyl active ester was prepared and subsequently conjugated to amino-C6-modified DNAs, resulting in GalNAc-Apc001 and other GalNAc-DNA derivatives. The acetyl groups on GalNAc were removed using ammonium hydroxide, and the final products were purified via high-performance liquid chromatography (HPLC) and their identity confirmed via electrospray ionization mass spectrometry (ESI-MS, Figure S1). The binding affinity of the conjugated aptamers was evaluated by using biolayer interferometry (BLI). Results showed that GalNAc-Apc001 exhibited a binding affinity of 32.5 nM toward sclerostin, comparable to that of unconjugated Apc001 (35 nM), indicating that GalNAc conjugation did not affect the interaction between Apc001 and sclerostin (Figure S2). The stability of GalNAc-Apc001 in 20% fetal bovine serum (FBS), snake venom phosphodiesterase (SVPDE), and SD rat liver microsomes (LMs) was markedly higher than that of the unmodified aptamer and comparable to that of Apc001 (Figure S3), suggesting its potential for *in vivo* applications.

Next, we assessed the ASGPR-mediated cellular uptake of GalNAc-aptamers (Figure S4a) using confocal microscopy and flow cytometry analysis. HepG2 cells, a hepatocarcinoma cell line with a high ASGPR expression, served as the positive control. A375 cells, a melanoma cell line lacking ASGPR expression, were used as the negative control. In HepG2 cells, treatment with GalNAc-Apc001-FAM and GalNAc-Scramble-FAM resulted in increased fluorescence signals, whereas Apc001-FAM treatment did not (Figure S4b,d), suggesting that the internalization of GalNAc-aptamer conjugates was GalNAc-dependent. In contrast, none of the treatments, including GalNAc-Apc001-FAM, GalNAc-Scramble-FAM, and Apc001-FAM, produced increased fluorescence in A375 cells (Figure S4c,e), indicating that the uptake of GalNAc-aptamers was ASGPR-dependent. Collectively, these findings demonstrated that the cellular internalization of GalNAc-aptamers required both GalNAc modification and ASGPR expression.

### GalNAc-Apc001-Facilitated Liver-Specific Internalization of Sclerostin and Bone-Targeted Accumulation In Vitro

To evaluate whether GalNAc-Apc001 can facilitate the liver-specific internalization of sclerostin (Figure 2a), cells were incubated in a culture medium containing a 1:1 mixture of aptamers (GalNAc-Apc001, GalNAc-Scramble, or Apc001) and FAM-labeled sclerostin (Sclerostin-FAM). Confocal microscopy and flow cytometry analyses revealed that GalNAc-Apc001 significantly increased the fluorescence signals in HepG2 cells (Figure 2b,c), while no detectable increase was observed in A375 cells (Figure S5). The fluorescence intensity was both concentration-dependent (Figure S6) and time-dependent (Figure 2d–f) in HepG2, indicating that GalNAc-Apc001 could facilitate the liver-specific internalization of sclerostin. In contrast, Apc001 produced no increased fluorescence in HepG2 cells (Figure 2b,c), suggesting that the liver-specific internalization of sclerostin requires the GalNAc moiety. Colocalization of the FAM signal with LysoTracker confirmed that the liver-specific internalization of sclerostin was rapidly transported to lysosomes. Furthermore, quantitative adsorption data revealed that GalNAc-Apc001 bound effectively to hydroxyapatite (HA) with a capacity of 75 μg/mg (w/w) (Figures 2g and S7). This high binding affinity indicated a strong potential for bone-specific accumulation and a prolonged pharmacokinetic half-life.

**Figure 2:**
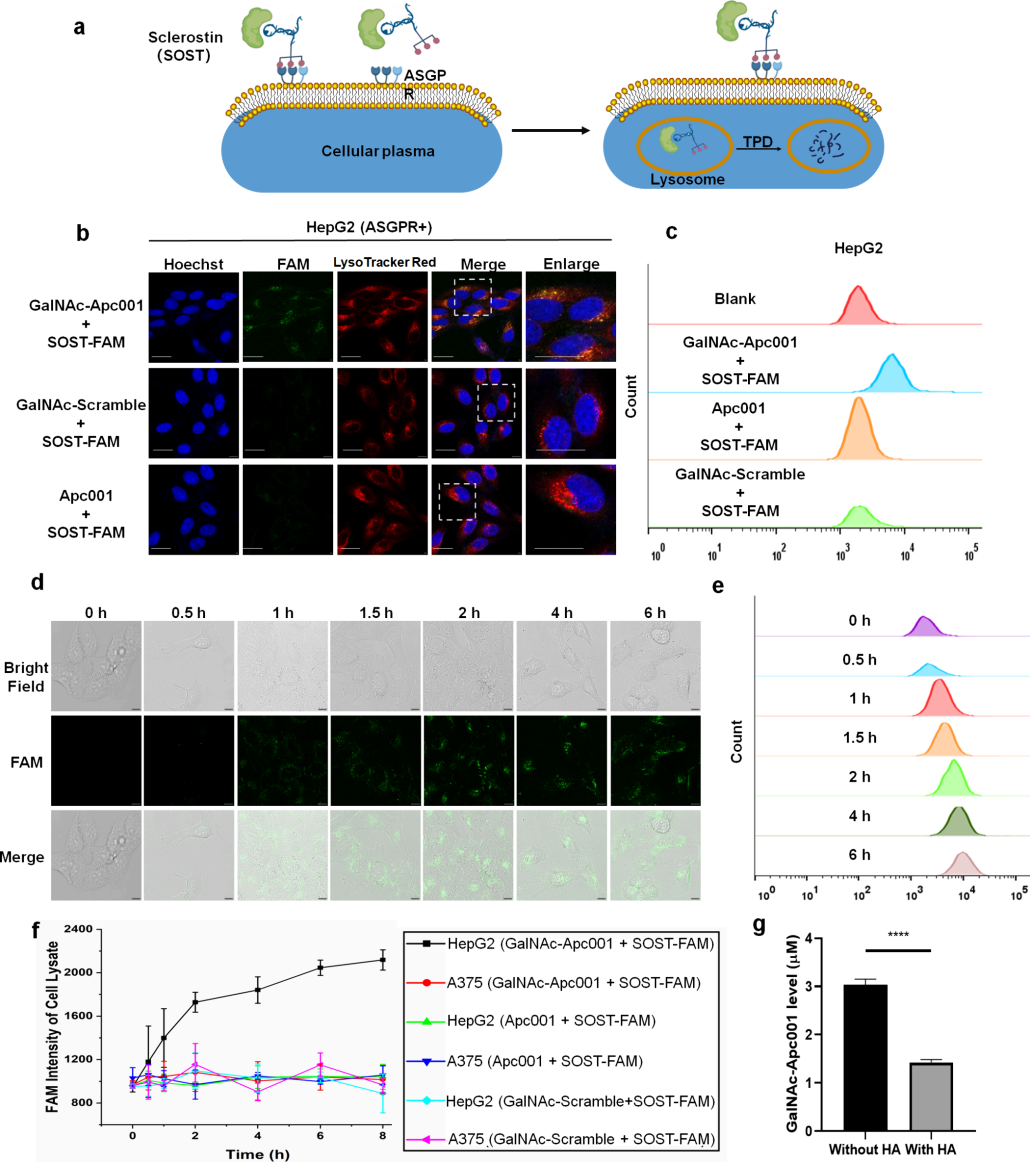
GalNAc-Apc001 facilitated the liver-specific internalization of sclerostin and bone-targeted accumulation *in vitro*. (a) Schematic illustration of GalNAc-Apc001-mediated ASGPR^+^ cell-specific internalization and lysosomal degradation of sclerostin. (b) The effects of GalNAc-Apc001, GalNAc-Scramble, or Apc001 on the endocytosis of SOST-FAM into ASGPR^+^ cells, detected by confocal microscopy. HepG2 cells were incubated for 2 h with 1 μM GalNAc-Apc001/SOST-FAM, GalNAc-Scramble/SOST-FAM, or Apc001/SOST-FAM. Scale bar: 25 μm. (c) The effects of GalNAc-Apc001, GalNAc-Scramble, or Apc001 on the cellular uptake of SOST-FAM into ASGPR^+^ cells, detected by flow cytometry analysis. HepG2 cells were incubated for 2 h with 1 μM GalNAc-Apc001/SOST-FAM, GalNAc-Scramble/SOST-FAM, or Apc001/SOST-FAM. (d) Time-dependent effects of GalNAc-Apc001 on the endocytosis of SOST-FAM into ASGPR^+^ cells detected by confocal microscopy. HepG2 cells were incubated for 0–6 h with 1 μM GalNAc-Apc001/SOST-FAM, GalNAc-Scramble/SOST-FAM, or Apc001/SOST-FAM. Scale bar: 10 μm. (e) The time-dependent effects of GalNAc-Apc001 on the cellular uptake of SOST-FAM into ASGPR^+^ cells, detected by flow cytometry analysis. HepG2 cells were incubated for 0–6 h with 1 μM GalNAc-Apc001/SOST-FAM. (f) The time-dependent effects of GalNAc-Apc001, GalNAc-Scramble, or Apc001 on the internalization of SOST-FAM into ASGPR^+^ cells or ASGPR^–^ cells, detected by microplate reader analysis. HepG2 cells or A375 cells were incubated for 0–8 h with 0.1 μM GalNAc-Apc001/SOST-FAM, GalNAc-Scramble/SOST-FAM, or Apc001/SOST-FAM. *n* = 3 per group. (g) The adsorption capacity of GalNAc-Apc001 in HA. HA was incubated for 3 h with 3 μM GalNAc-Apc001. *n* = 3 per group. Notes: Data were presented as the mean ± standard deviation (SD). Statistical analysis was performed using an unpaired *t* test: ***P < 0.001. SOST indicated sclerostin; FAM indicated fluorescein amidite; HA indicated hydroxyapatite; GalNAc-Apc001/SOST-FAM, GalNAc-Scramble/SOST-FAM, or Apc001/SOST-FAM indicated GalNAc-Apc001, GalNAc-Scramble, or Apc001 was premixed with SOST-FAM in a 1:1 ratio. Nuclei were stained with Hoechst 33342 (blue), SOST-FAM were labeled with FAM (green), and lysosomes were stained with LysoTracker Red.

To investigate the internalization pathway, HepG2 cells were treated with a 1:1 mixture of GalNAc-Apc001 and sclerostin, with and without chlorpromazine (clathrin-endocytosis inhibitor), amiloride (micropinocytosis inhibitor), or genistein (caveolae-endocytosis inhibitor) pretreatment.^39^ Confocal microscope results showed that chlorpromazine completely blocked the internalization of sclerostin-FAM (Figure S8a,b), suggesting a clathrin-dependent pathway. To validate this, we pretreated HepG2 cells with chlorpromazine before adding a 1:1 mixture of GalNAc-Apc001 and sclerostin. Western blot analysis confirmed that chlorpromazine abolished GalNAc-Apc001-mediated sclerostin endocytosis (Figure 3a), further supporting the role of clathrin-mediated uptake. This finding was consistent with previous reports.^40^

**Figure 3:**
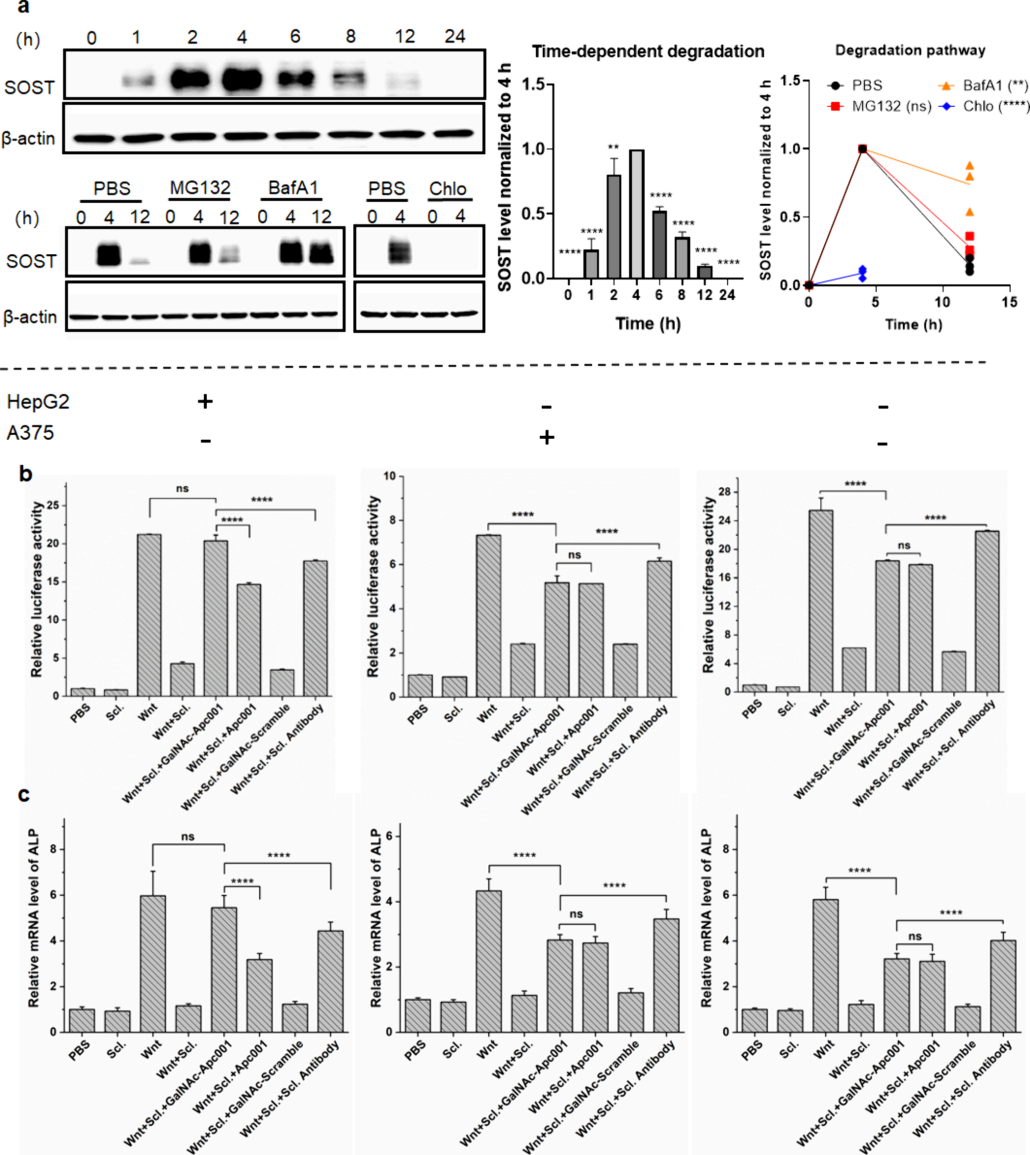
GalNAc-Apc001 induced time- and lysosome-dependent degradation of sclerostin, alleviated its inhibition of Wnt signaling, and promoted bone formation marker expression in the presence of ASGPR^+^ cells. (a) The time- and lysosome/clathrin-dependent effects of GalNAc-Apc001 on the degradation of SOST in ASGPR^+^ cells, detected by Western blot analysis. For 0–4 h, HepG2 cells were incubated with DMEM medium containing 1 μM GalNAc-Apc001/SOST with and without lysosome/clathrin inhibitors. At the time point of 4 h, the medium was exchanged with a normal DMEM medium. β-Actin was utilized as an internal control. (b) The inhibitory effects of GalNAc-Apc001, Apc001, GalNAc-Scramble, or Scl. Antibody on the SOST-suppressed Wnt signaling in HEK293 cells cocultured with HepG2 cells or A375 cells, detected by a TOPFlash reporter assay. *n* = 3 per group. (c) The effects of GalNAc-Apc001, Apc001, GalNAc-Scramble, or Scl. Antibody on the upregulation of the mRNA expressions of bone formation marker (*Alp*) in MC3T3-E1 cells cocultured with HepG2 cells or A375 cells, detected by an RT-qPCR assay. *n* = 3 per group. Notes: Data were normalized to the PBS control group and presented as the mean ± SD. Statistical significance was determined using one-way ANOVA followed by Tukey’s post hoc test: ^ns^ *P* > 0.05, **P < 0.01, ***P < 0.001, and ****P < 0.0001. SOST indicated sclerostin; GalNAc-Apc001/SOST indicated GalNAc-Apc001 was premixed with SOST at a 1:1 ratio; MG132 was a proteasome inhibitor; BafA1 indicated bafilomycin A1 (lysosome inhibitor); Chlo indicated chlorprothixene (clathrin inhibitor); PBS indicated groups treated with 1× PBS (volume matched to other treatments); Scl indicated groups treated with 100 nM sclerostin; Wnt indicated groups transfected with Wnt-1 plasmid; and Scl-Antibody indicated groups treated with sclerostin-targeting antibody (romosozumab).

### GalNAc-Apc001 Induced Time- and Lysosome-Dependent Degradation of Sclerostin, Alleviated Its Inhibition of Wnt Signaling, and Promoted Bone Formation Marker Expression in the Presence of ASGPR^+^ Cells

To investigate whether GalNAc-Apc001 can specifically induce the degradation of extracellular sclerostin in ASGPR-positive cells in a time- and lysosome-dependent manner, HepG2 cells were treated with a 1:1 mixture of GalNAc-Apc001 and sclerostin for 0–4 h, with and without MG132 (proteasome inhibitor) or Bafilomycin A1 (lysosome inhibitor) pretreatment. At the time point of 4 h, the medium was exchanged with normal DMEM medium. Western blot analysis showed an increase in the sclerostin level in HepG2 cells over the first 4 h. Upon replacement with fresh medium, accumulated sclerostin was gradually degraded over the following 20 h (4–24 h). Notably, Bafilomycin A1 instead of MG132 could reverse the degradation of sclerostin in HepG2 within 12 h (Figure 3a). Collectively, these results strongly supported the notion that GalNAc-Apc001 mediated ASGPR-specific internalization and lysosomal degradation of extracellular sclerostin. Additionally, GalNAc-Apc001 exhibited no cytotoxicity in various liver cell lines (HepG2 and Hepa1–6) at concentrations ranging from 0.4 to 6.6 μM, demonstrating a favorable safety profile (Figure S9).

To investigate the effects of GalNAc-Apc001 on Wnt signaling suppressed by sclerostin, HEK293 cells, widely used in Wnt signaling research, were cotransfected with three plasmids: a firefly luciferase reporter plasmid under the transcriptional control of a TCF/LEF response element (activated by Wnt signaling), an SV40 plasmid encoding renilla luciferase (internal control),^41^ and a Wnt-1 expression plasmid. After 6 h, the medium was replaced with fresh medium containing sclerostin to establish a model of sclerostin-induced Wnt signaling suppression. In this model, treatment with sclerostin inhibitors is expected to restore Wnt signaling, as indicated by the reactivation of firefly luciferase expression.^42^ The transfected HEK293 cells were cocultured in transwells with additional cells (HepG2, A375, or no cells) and treated with various agents: GalNAc-Apc001, Apc001, GalNAc-Scramble, or Scl-Antibody. After 12 h, cells were lysed, and the luciferase activity was measured using a dual-reporter assay. As shown in Figure 3b, in the presence of HepG2 cells, GalNAc-Apc001 restored relative luciferase activity to 96%, which was 39% higher than that achieved with Apc001. However, when HepG2 cells were replaced with A375 cells or omitted entirely, no significant difference was observed between the GalNAc-Apc001 and Apc001 treatment groups. Additionally, GalNAc-Scramble failed to restore luciferase activity under any condition. These results indicated that the enhanced reactivation of Wnt signaling by GalNAc-Apc001 depended on the ASGPR-mediated uptake of sclerostin. Notably, GalNAc-Apc001 demonstrated a significantly stronger reactivation effect than that of Scl-Antibody in the presence of HepG2 cells, highlighting its therapeutic potential.

To further evaluate the effects of GalNAc-Apc001 on bone formation, MC3T3-E1 cells (preosteoblast-like cells) were used to measure the expression of bone formation markers (*Alp*, *Runx2*, and *Ocn*) suppressed by sclerostin. These cells were first transfected with the Wnt-1 plasmid for 6 h and then cocultured with HepG2 cells, A375 cells, or no cells in fresh medium. Sclerostin protein and the same set of treatments (GalNAc-Apc001, Apc001, GalNAc-Scramble, or Scl-Antibody) were added, and mRNA levels of *Alp* (Figure 3c), *Runx2* (Figure S10a), and *Ocn* (Figure S10b) were quantified via real-time qPCR. Consistent with the luciferase assay, in the presence of HepG2 cells, GalNAc-Apc001 restored *Alp*, *Runx2*, and *Ocn* mRNA levels to levels comparable to those of untreated controls. Compared to Apc001, GalNAc-Apc001 increased the mRNA expression by 1.5–1.9 fold (71% for *Alp*, 52% for *Runx2*, and 92% for *Ocn*). In contrast, no significant differences were observed between GalNAc-Apc001 and Apc001 treatments in the presence of A375 cells or without coculture. Furthermore, GalNAc-Scramble did not increase the marker expression under any condition. Importantly, GalNAc-Apc001 showed a significantly greater upregulation of *Alp*, *Runx2*, and *Ocn* mRNA levels than that of Scl-Antibody when HepG2 cells were present. Conversely, in the absence of HepG2 cells, its effect was significantly lower than that of Scl-Antibody. Together, these findings confirmed that GalNAc-Apc001 promoted bone formation *in vitro* through an ASGPR^+^ cell-mediated mechanism.

### GalNAc-Apc001 Facilitated Liver-Specific Recognization and Bone-Targeted Accumulation In Vivo

To evaluate the impact of GalNAc conjugation on the biodistribution of Apc001, a tissue distribution study was conducted using biophotonic imaging to measure the Cy3 fluorescence intensity across major organs in C57BL/6 mice. Six- to eight-week-old mice were subcutaneously administered PBS (Vehicle), Cy3-labeled Apc001 (Apc001), Cy3-labeled GalNAc-Apc001 (GalNAc-Apc001), or the Cy3-labeled scrambled sequence (Scramble). At 2 h postinjection, fluorescence signals in the liver and bone were significantly higher in mice treated with GalNAc-Apc001 compared to those treated with Apc001 or Scramble (Figure 4a). Notably, fluorescence was barely detectable in the heart, spleen, and lungs across all groups. Quantitative analysis of the percentage of the injected dose in major organs confirmed that GalNAc-Apc001 exhibited markedly greater liver and bone accumulation in comparison to either Apc001 or Scramble (Figure 4a).

**Figure 4:**
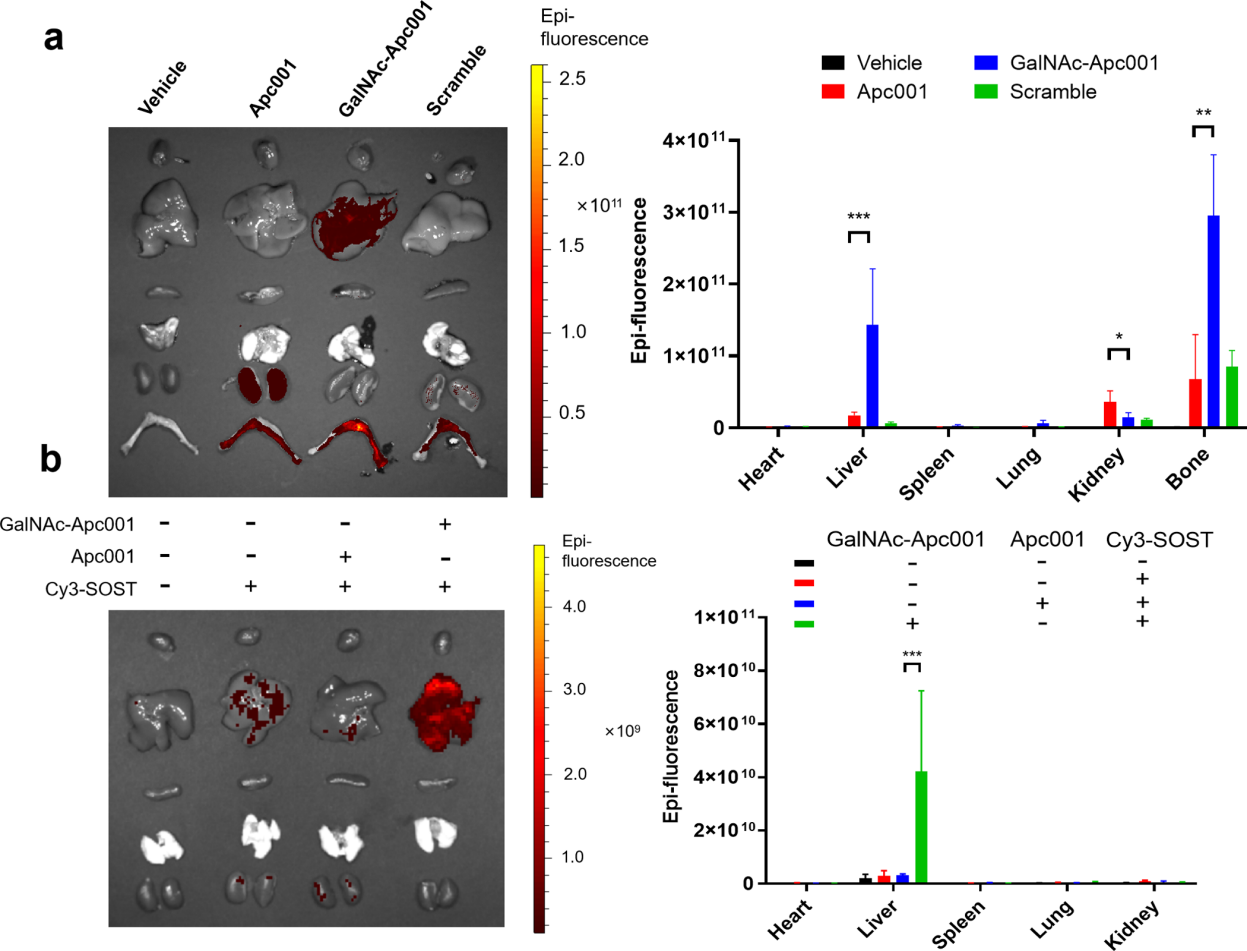
GalNAc-Apc001 facilitated liver-specific recognization and bone-targeted accumulation *in vivo*. (a) The biodistribution of Cy3-labeled Apc001, GalNAc-Apc001, or Scramble in heart, liver, spleen, lung, kidney, and bone isolated from C57BL/6 mice, detected by small animal *in vivo* imaging assay and statistical analysis. The mice received a subcutaneous injection of the indicated groups (10 nmol per mouse) for 2 h. *n* = 4 mice per group. (b) The effect of Cy3-labeled Apc001, GalNAc-Apc001, or Scramble on the delivery of sclerostin to heart, liver, spleen, lung, and kidney from C57BL/6 mice, detected by small animal *in vivo* imaging assay and statistic analysis. The mice pretreated with 1 nmol Cy3-labeled recombinant sclerostin protein via intraperitoneal injection for 1 h, followed by subcutaneous injection of the indicated groups (10 nmol per mouse) for 2 h. *n* = 3 mice per group. Notes: Data were presented as the mean ± SD. Statistical significance was determined using an unpaired *t* test: *P < 0.05, **P < 0.01, and ***P < 0.001. Cy3 indicates cyanine-3 fluorescent dye.

To further assess the ability of GalNAc-Apc001 to direct sclerostin to liver tissue, a second tissue distribution study was performed. C57BL/6 mice, with or without pretreatment using Cy3-labeled sclerostin, were subcutaneously administered PBS, Apc001, or GalNAc-Apc001. In mice pretreated with Cy3-labeled sclerostin, only minimal sclerostin accumulation was observed in the liver. However, when GalNAc-Apc001 was administered, the liver fluorescence intensity increased significantly compared to that of the Apc001-treated group at 2 h postinjection (Figure 4b). Again, fluorescence signals in the heart, spleen, and lungs remained negligible. Quantitative analysis of the Cy3-labeled sclerostin distribution confirmed that GalNAc-Apc001 significantly enhanced the liver-specific delivery of sclerostin compared to Apc001 (Figure 4b). These findings demonstrated that GalNAc-Apc001 could be rapidly and preferentially delivered to liver tissue, potentially minimizing off-target distribution and renal clearance. Moreover, the data provide compelling evidence that GalNAc-Apc001 effectively mediated the liver-specific delivery of sclerostin *in vivo*.

### GalNAc-Apc001 Promoted Bone Formation, Increased Bone Mass, Enhanced Bone Mechanical Properties, and Improved Bone Architecture in Osteogenesis Imperfect Mice

To assess the *in vivo* efficacy of GalNAc-Apc001, we first conducted a comprehensive toxicity evaluation of the agents used. Six- to eight-week-old osteogenesis imperfecta (OI) mice received weekly subcutaneous injections for 12 weeks with one of the following treatments: PBS (OI-Vehicle), carboxylic Apc001 at 25 mg·kg^–1^ (Apc001), GalNAc-Apc001 at 25 mg·kg^–1^, or sclerostin antibody at 25 mg·kg^–1^ (Scl-Antibody). Wild-type mice were similarly treated with PBS (WT-Vehicle), and *Col1a2* ^*+/G610C*^ mice were sacrificed prior to treatment to serve as the OI-Baseline group (Figure 5a).

**Figure 5:**
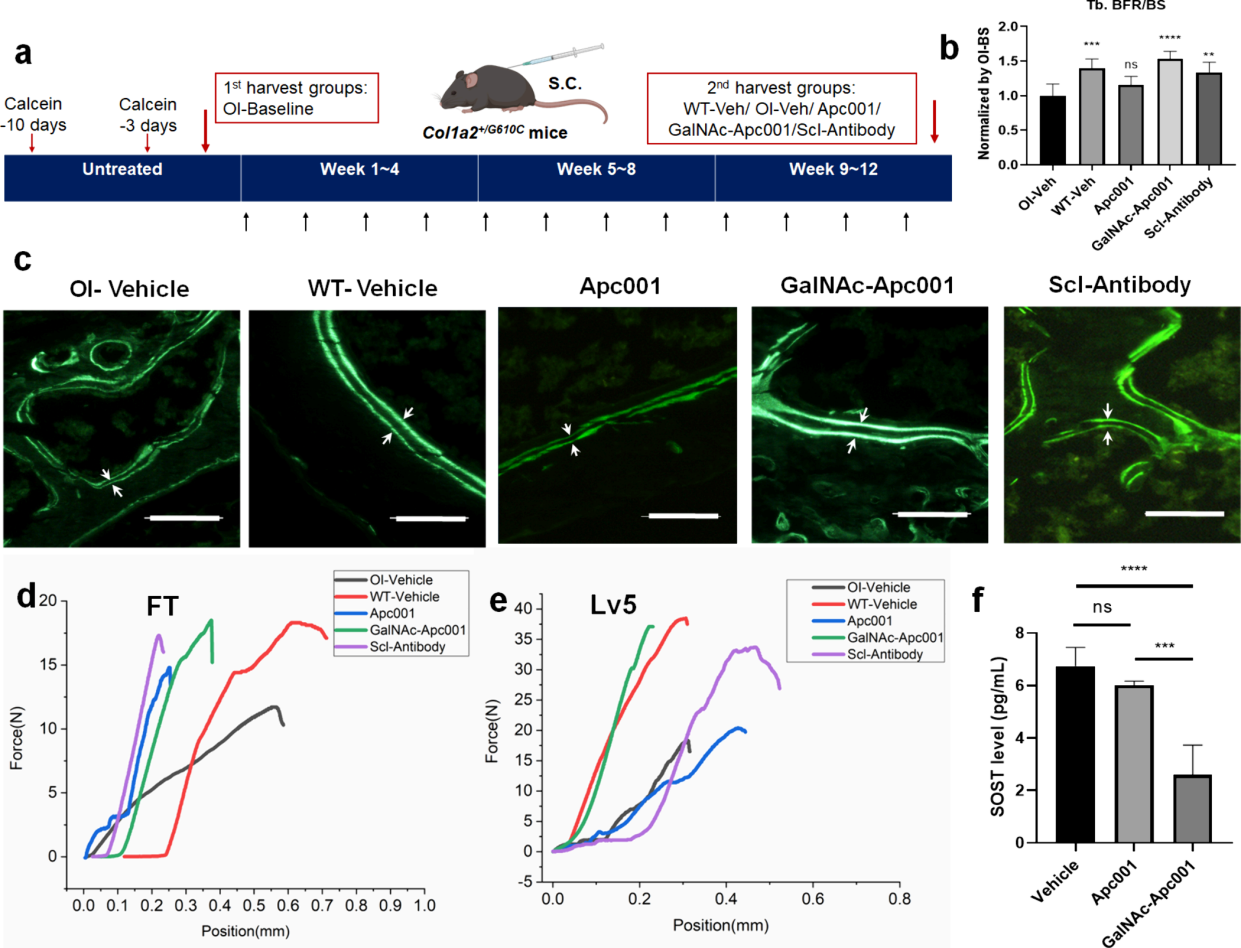
GalNAc-Apc001 promoted bone formation and improved bone mechanical properties in *Col1a2* ^*+/G610C*^ OI mice. (a) Schematic illustration of the *in vivo* experimental design. (b) Bar graph showing the effects of OI-Veh, WT-Veh, Apc001, GalNAc-Apc001, or Scl-Antibody on the upregulation of the dynamic bone histomorphometric parameters of Tb.BFR/BS at the distal femur. *n* = 6 per group. (c) Representative fluorescent micrographs of the trabecular bone sections showing the effects of the indicated groups on the bone formation at the distal femur visualized by calcein green labels. *n* = 6 per group. Scale bars: 0.1 mm. (d) Representative curves showing the effects of the indicated groups on the bone mechanical properties of the distal femur, detected by a three-point bending test. *n* = 6 per group. (e) Representative curves showing the effects of the indicated groups on the bone mechanical properties of the fifth lumbar vertebrae (Lv5), detected by a compression test. *n* = 6 per group. (f) Bar graph showing the effects of Apc001 or GalNAc-Apc001 on the downregulation of serum sclerostin levels, detected by ELISA assay. *n* = 4 per group. Notes: Data were normalized by the parameters in OI baseline groups and presented as the mean ± SD. Statistical significance was determined using one-way ANOVA with Tukey’s posthoc test. ^ns^ *P* > 0.05, **P < 0.01, ***P < 0.001, and ****P < 0.0001. Tb.BFR/BS indicates the trabecular bone formation rate per unit of bone surface; OI-BS indicates OI-Baseline; OI-Veh indicates OI-Vehicle; WT-Veh indicates WT-Vehicle; Apc001 indicates carboxylic Apc001 (25 mg·kg^–1^); GalNAc-Apc001 indicates GalNAc conjugated Apc001 (25 mg·kg^–1^); Scl-Antibody indicates sclerostin antibody (25 mg·kg^–1^); and SOST indicates sclerostin.

Histological analysis using hematoxylin and eosin staining revealed polygonal or rhombic cells with spindle-shaped or large spherical nuclei across all groups, indicating normal cellular morphology and minimal apoptosis (Figure S11). To further investigate potential inflammatory responses in liver tissue, immunostaining for IL-6 and TNF-α, common markers of inflammation, was performed. The GalNAc-Apc001 group exhibited comparable levels of IL-6 and TNF-α-positive cells to the WT-Vehicle, OI-Vehicle, and Apc001 groups and notably lower levels than the Scl-Antibody group, suggesting minimal inflammatory response following GalNAc-Apc001 treatment (Figure S11). Biochemical analysis of liver and kidney function parameters showed no significant differences between treatment groups and the WT-Vehicle or OI-Vehicle controls, except for alanine aminotransferase (ALT). The Scl-Antibody group displayed significantly elevated ALT levels compared to both control groups (Figure S12). These findings support the favorable safety profile of GalNAc-Apc001 as a therapeutic candidate for the OI.

To evaluate the anabolic effects of GalNAc-Apc001 on bone, histomorphometric analysis was performed to measure trabecular bone formation at the distal femur in *Col1a2* ^*+/G610C*^ mice. After normalization to the OI-Baseline group, the trabecular bone formation rate (Tb.BFR/BS) was calculated. Compared to the OI-Vehicle group, Tb.BFR/BS was increased by 1.40-fold in the WT-Vehicle group (*P* < 0.001), 1.16-fold in the Apc001 group (*P* > 0.05), 1.53-fold in the GalNAc-Apc001 group (*P* < 0.0001), and 1.33-fold in the Scl-Antibody group (*P* < 0.01). No significant difference was observed between the WT-Vehicle and GalNAc-Apc001 groups, indicating that GalNAc-Apc001 restored bone formation in OI mice to near-normal levels (Figure 5b,c).

To assess the bone mechanical strength, a three-point bending test was conducted on the distal femur. Compared to the OI-Vehicle group, the failure force increased by 1.56-fold in the WT-Vehicle group, 1.26-fold in the Apc001 group, 1.58-fold in the GalNAc-Apc001 group, and 1.45-fold in the Scl-Antibody group. After 12 weeks of GalNAc-Apc001 treatment at 25 mg·kg^–1^, the mechanical strength of the distal femur in OI mice was comparable to that of wild-type mice (Figure 5d). Additionally, a compression test was performed on the fifth lumbar vertebra. Compared to the OI-Vehicle group, the failure force increased by 2.10-fold in the WT-Vehicle group, 1.11-fold in the Apc001 group, 2.03-fold in the GalNAc-Apc001 group, and 1.59-fold in the Scl-Antibody group. Consistently, GalNAc-Apc001 treatment restored vertebral strength in OI mice to levels comparable to wild-type controls (Figure 5e). Notably, an ELISA revealed that serum sclerostin levels were significantly reduced following GalNAc-Apc001 treatment (Figure 5f), indicating its therapeutic efficacy in lowering circulating sclerostin.

To evaluate the bone anabolic effects of GalNAc-Apc001 in osteogenesis imperfecta (OI) mice, microcomputed tomography (micro-CT) was employed to assess the trabecular bone mass and microarchitecture at the metaphysis of the distal femur in *Col1a2* ^*+/G610C*^ mice. After normalization to the OI-Baseline group, the following parameters were calculated: trabecular connect density (Tb. conn.D), trabecular volumetric mineral density (Tb. vBMD), and trabecular bone volume fraction (Tb. BV/TV) for evaluating the trabecular bone mass were calculated. The trabecular number (Tb. N), trabecular thickness (Tb. Th), and trabecular separation (Tb. Sp) for evaluating the trabecular microarchitecture were calculated. For the distal femur, the micro-CT results indicated that the GalNAc-Apc001 group had remarkably higher Tb. conn.D (+95%, *P* < 0.05), Tb. vBMD (+167%, *P* < 0.0001), Tb. BV/TV (+120%, *P* < 0.01), Tb. N (+71%, *P* < 0.01), Tb. Th (+42%, *P* < 0.01), and Tb. Sp (−46%, *P* < 0.01) compared to those of the OI-Vehicle group, which were also significantly higher than for the Apc001-OA group and Scl-Antibody group (Figure 6a,b).

**Figure 6:**
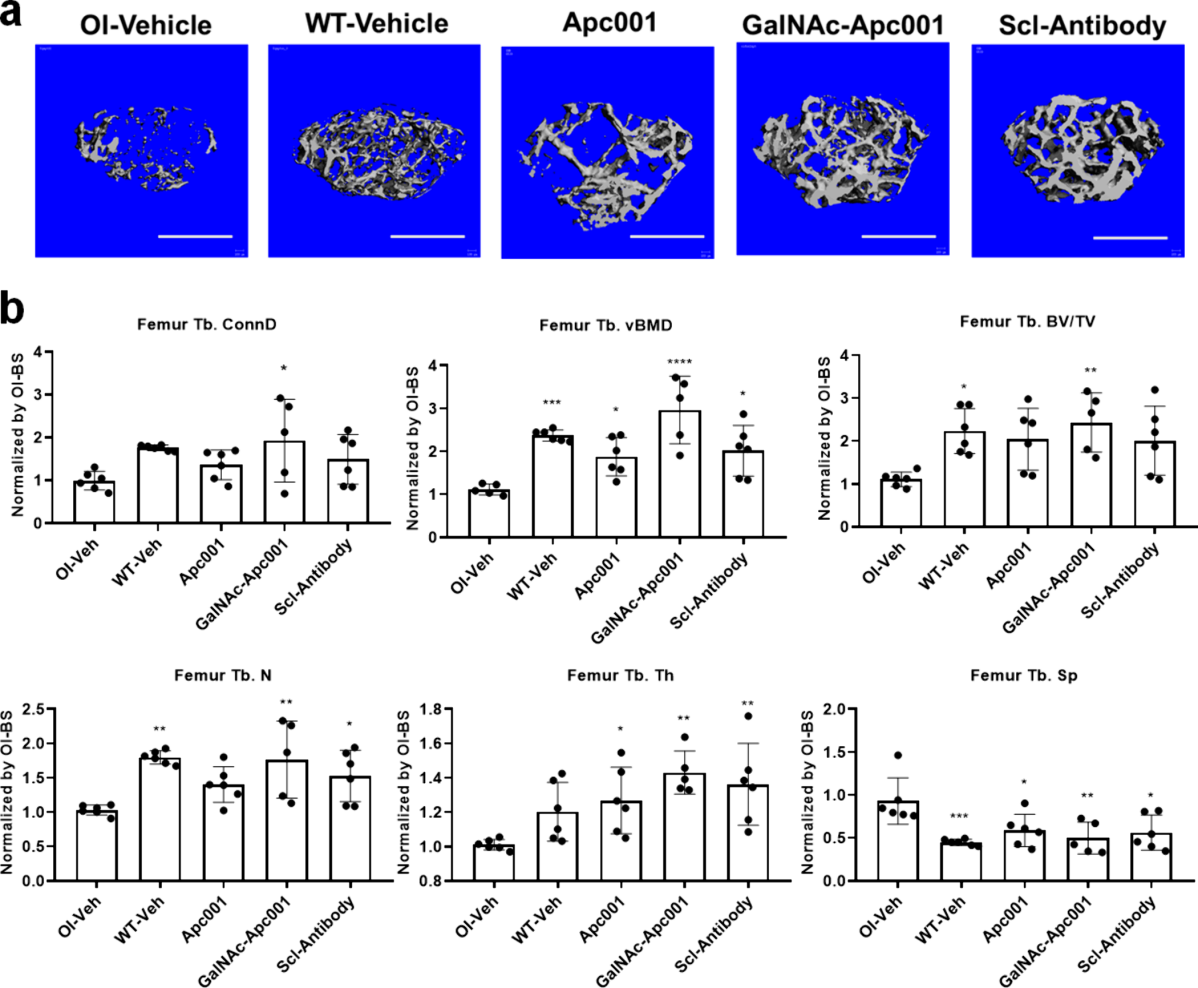
GalNAc-Apc001 increased trabecular bone mass and improved trabecular microarchitecture of the distal femur in *Col1a2* ^*+/G610C*^ OI mice. (a) Representative image showing the effects of OI-Veh, WT-Veh, Apc001, GalNAc-Apc001, or Scl-Antibody on the three-dimensional trabecular architecture at the distal femur, detected by micro-CT reconstruction. *n* = 6 per group. Scale bars, 1 mm. (b) Bar graphs showing the effects of the indicated groups on the structural parameters of Tb. conn.D, Tb. vBMD, Tb.BV/TV, Tb. N, Tb.Th, and Tb.Sp at the trabecular bone of the proximal tibia, detected by *ex vivo* micro-CT examination. *n* = 6 per group. Notes: Data were normalized by the parameters in OI baseline groups and presented as mean ± SD. Statistical significance was determined using one-way ANOVA with Tukey’s posthoc test. *P < 0.05, **P < 0.01, ***P < 0.001, and ****P < 0.0001. Tb.conn.D indicates the trabecular connectivity density; Tb.vBMD indicates the trabecular volumetric bone mineral density; Tb.BV/TV indicates the trabecular volume per total volume; Tb.N indicates the trabecular number; Tb.Th indicates the trabecular thickness; Tb.Sp indicates the trabecular spacing; OI-BS indicates OI-Baseline; OI-Veh indicates OI-Vehicle; WT-Veh indicates WT-Vehicle; Apc001 indicates carboxylic Apc001 (25 mg·kg^–1^); GalNAc-Apc001indicates GalNAc-conjugated Apc001 (25 mg·kg^–1^); and Scl-Antibody indicates sclerostin antibody (25 mg·kg^–1^).

Collectively, GalNAc-Apc001 could promote bone formation, increase bone mass, enhance bone mechanical properties, and improve bone architecture in osteogenesis imperfect mice (*Col1a2* ^*+/G610C*^ mice).

## Conclusions

Extracellular targeted protein degradation (eTPD) engaging the asialoglycoprotein receptor (ASGPR) has recently emerged as a promising therapeutic modality for targeting extracellular proteins, offering a novel mechanism of action and a favorable safety profile. However, ASGPR-mediated eTPD has not yet been explored for the treatment of bone diseases. In this study, we engineered a bone-homing sclerostin aptamer (Apc001) into an aptamer-based eTPD molecule by conjugating a tri-GalNAc moiety to its 5′-terminus, resulting in GalNAc-Apc001. This design enables hepatocyte-specific uptake via ASGPR and the subsequent lysosomal degradation of sclerostin. Our *in vitro* and *in vivo* data collectively demonstrated that GalNAc-Apc001 exhibited superior therapeutic efficacy compared to both the parent aptamer and the marketed sclerostin antibody. Mechanistic investigations revealed that the enhanced efficacy of GalNAc-Apc001 was attributed to its optimized biodistribution and liver-specific degradation of sclerostin. These findings highlighted the potential of aptamer-based eTPD as a transformative platform for aptamer drug development. Compared to antibody-based eTPD technologies, aptamer-based approaches offer distinct advantages in industrial scalability due to their simplicity in chemical manufacturing and control (CMC). While these results are encouraging, further studies are warranted to fully elucidate the long-term safety, pharmacokinetics, and translational potential of aptamer-based eTPD strategies in clinical settings.

## Supplementary Material



## Data Availability

All data supporting the findings of this study are available within the article and its Supporting Information. Additional data is available from the corresponding author upon reasonable request.

## Notes

Supplementary figures and tables, materials and methods, details of statistical tests, detailed synthetic procedures and compound ESI MS analyses, biolayer interferometry analyses, confocal microscopy images, flow cytometry analysis, qPCR, hematoxylin-eosin staining and immunohistochemical staining analysis, and the liver and kidney functions parameters (PDF)
