# Supplementary material for: Harnessing Bone-Liver Crosstalk: A Dual-Action LYTAC Approach for Bone-Specific Accumulation and Liver-Specific Protein Degradation in Bone Disorders
Source: JACS Au. 2025 Nov 20;5(12):5973–84. doi: 10.1021/jacsau.5c00827 (PMC12728634; doi:10.1021/jacsau.5c00827)
Supplement: Supplementary file 1 [file au5c00827_si_001.pdf]

Supporting Information

Harnessing Bone-Liver Crosstalk: A Dual-Action LYTAC Approach for Bone-Specific Accumulation and Liver-Specific Protein Degradation in Bone Disorders

Yuan Ma[a,b,||], Gubu Amu[c,||], Yufei Pan[c,||], Hewen Jiang[a,||], Sifan Yu[b], Huarui Zhang[a], Zefeng Chen[b], Hang Luo[a], Chuanxin Zhong[b], Xin Yang[b], Xiaohui Tao[b], Yihao Zhang[a], Yuanyuan Yu[b], Aiping Lu[b], Luyao Wang[b,*], Baoting Zhang[a,*], Ge Zhang[b,*]

[a] *School of Chinese Medicine, Faculty of Medicine, The Chinese University of Hong Kong, New Territories, Hong Kong SAR 999077, China.*

[b] *Law Sau Fai Institute for Advancing Translational Medicine in Bone &Joint Diseases, School of Chinese Medicine, Hong Kong Baptist University, Kowloon City, Hong Kong SAR 999077, China.*

[c] *Aptacure Therapeutics Limited, New Territories, Hong Kong SAR 999077, China.*

[||]Y.M., G.A. Y.P., and H.J. contributed equally.

*Corresponding authors: luyaowang@hkbu.edu.hk (L.W.); zhangbaoting@cuhk.edu.hk (B.Z.); zhangge@hkbu.edu.hk (G.Z.)

Experimental section

Materials

The nucleic acids used in this work were synthesized using AKTA Oliogopilot 100 Oligonucleotide Synthesizer (GE Healthcare, USA). The recombinant proteins were purchased from Abcam (UK). The other chemical reagents were purchased from Aldrich Sigma (USA). The MC3T3-E1, HEK293, A375, HepG2 cells were purchased from ATCC (USA). TrypanBlue (A514-100) was purchased from BDBIO (HangZhou, China). 1×Hoechst 33342 staining solution (C1025) and Lysotracker Red (C1046) were purchased from Beyotime (China). The plasmids used in this study were purchased from GenScript Biotech Corporation (USA). Fetal Bovine Serum (A5670701), Dulbecco's modified Eagle's medium (11965092), MEM-α medium (12571063) and OptiMem Medium (31985062) were purchased from Gibco (USA). Lipofectamine 3000 (L3000015) was purchased from Invitrogen (USA). The OI mice (*Col1a2*$^{+/G610C}$ mice) and wild-type mice were purchased from the Laboratory Animal Services Centre in the Chinese University of Hong Kong (LASEC, CUHK, HK). Penicillin-Streptomycin (211092) was purchased from NEST Biotechnology Co. Ltd. (Wuxi, China). Lysis Buffer (A8261) and Dual-Luciferase Reporter Assay System (E1910) were purchased from Promega (USA). The genistein (ST00650120) was purchased from Shanghai Standard Technology Co.,Ltd (China). EZ-Link™ NHS-PEG4 Biotinylation Kit (21455) was purchased from Thermo Scientific (USA). TransZol Up Plus RNA Kit (ER501-01) was purchased from TransGen Biotech (China). Tri-GalNAc carboxylic acid derivative was purchased from WuXi AppTec (China).

Preparation of GalNAc-Apc001 conjugates

N-hydroxysuccinimide (NHS) (50.7 mg, 0.45 mmol) and 1-ethyl-3-(3-dimethylaminopropyl) carbodiimide (EDCI) (85.7 mg, 0.45 mmol) were added to the stirred solution of tri-GalNAc carboxylic acid derivative (compound 1) (500 mg, 0.3 mmol) in anhydrous dichloromethane (DCM) (20 mL) under nitrogen. The mixture was left standing overnight, and subsequently halted by adding the saturated $NaHCO_3$. The remaining mixture was quickly partitioned using DCM (100 mL) and saturated $NaHCO_3$ (60 mL). The organic layer was then washed with saturated NaCl, dried with anhydrous Na_2SO_4, and finally concentrated to yield a crude product (compound 2) (540 mg, 0.246 mmol, yield ~82%). To a solution of amino-DNAs in triethylamine acetate (TEAA) (1.0 M, 5 mL, pH=8.0), compound 2 without further operation was added and left standing overnight. Then, the mixture was added to a solution of ammonium

hydroxide (10 mL) and heated to 60 °C for 3 h. After evaporated, the remaining residue was re-dissolved in dH_2O (1 mL). Afterwards, the mixture was purified using high-performance liquid chromatography (Agilent 1260). Phase A was acetonitrile (ACN), and phase B was TEAA (50 mM). The gradient was run from 5% to 60% of phase A in 30 min for the Xbride ®Oligonucleotide BEH C18 OBDTM Prep Column (2.5 μm, 10 mm×50 mm) with ambient column temperature at a flow rate of 1.2 $mL·min^{-1}$. The purified GalNAc-Apc001 (or GalNAc-scApc001, negative control) were desalted by using a Sephadex G25 column. The samples were further lyophilized to dryness for storage and confirmed by ESI MS analyses (Table S1).

Biolayer interferometry (BLI) assay

Corresponding aptamers, including Apc001 [C(OMe)G(OMe)G(OMe)G(OMe)GTGTGGGTTCGTCGTTAGCTTGATTTGGCAGCU(OMe)G(OMe)C(OMe)C(OMe)-idT] and Scramble [A(OMe)T(OMe)G(OMe)C(OMe)AAGCACATTGTGATCGCTTCAAATGTCTTCCGU(OMe)C(OMe)C(OMe)G(OMe)-idT] were used in the study. The binding affinity of Apc001, GalNAc-Apc001, GalNAc-Scramble and Scl-Antibody against sclerostin was measured by Octet 96/96e system (ForteBio) at 25 °C. Sclerostin was firstly biotinylated as the loading sample following the instructions of EZ-Link™ NHS-PEG4 Biotinylation Kit. The three nucleic acids were involved as the analysts at varied concentrations. Running processes and conditions were as follows: Baseline: 1×PBS buffer (2.7 mM KCl, 10 mM phosphate buffer, and 137 mM NaCl, pH 7.4), 120 s, 1000 rpm; Loading: 100 nM biotinated sclerostin in 1×PBST buffer (1×PBS + 0.02% Tween20, pH 7.4), 600 s, 500 rpm; Baseline 2: 1×PBST buffer, 300 s, 500 rpm; Association: Aptamers with varied concentrations in 1×PBST buffer, 500 rpm; Dissociation: 1×PBST buffer, 500 rpm; Regenration: 5 M NaCl, 30 s, 500 rpm; Neutralization: 1×PBS buffer, 60 s, 500 rpm. Finally, the affinity constant K_D values were calculated by fitting curves into a 1:1 binding model with ForteBio Data analysis 11.0.

Confocal laser scanning microscopy (CLSM) imaging assay

HepG2 and A373 cells were seeded onto a confocal plate at a density of $8×10^4$ cells in 2 mL DMEM medium. The cells were then cultured at 37 °C in a humidified atmosphere containing 5% CO_2 for a period of 24 h. Then preset concentration of GalNAc-Apc001-FAM, Apc001-FAM, GalNAc-Scramble, GalNAc-Apc001/Sclerostin-FAM mixture (1:1), Apc001/Sclerostin-FAM mixture (1:1), or GalNAc-Scramble/Sclerostin-FAM mixture (1:1) was added in the medium and incubated for a preset time. After that, the cells were stained by 1×Hoechst 33342

staining solution for nuclei and 50 nM Lysotracker Red for lysosomes at 37 °C for 30 min simultaneously. Wash the cells gently three times using 1×PBS buffer and keep the cells in 1×PBS buffer during the whole imaging process. The fluorescent imaging of the cells was performed with selected channels (Blue channel: 405 nm; Green channel: 488 nm; Red channel: 640 nm) at 60× water immersion mode using confocal laser scanning microscopy (LEICA TCS SP8).

For internalization pathways, HepG2 cells were seeded onto confocal plates at a density of 8×10^4 cells in 2 mL DMEM medium. The cells were then cultured at 37 °C in a humidified atmosphere containing 5% CO_2 for a period of 24 h. Then, the cells were respectively pre-treated with 1 mM Amiloride, 30 μM Chlorpromazine, 500 μM Genistern for 10 mimutes, followed by treatment with 1 μM GalNAc-Apc001/Sclerostin-FAM mixture (1:1) for 2 h. Next, the cells were stained by 1×Hoechst 33342 staining solution for nuclei and 50 nM Lysotracker Red for lysosomes at 37 °C for 30 minutes simultaneously. Wash the cells gently three times using 1×PBS buffer and keep the cells in 1×PBS buffer during the whole imaging process. The fluorescent imaging of the cells was performed with selected channels (Blue channel: 405 nm; Green channel: 488 nm; Red channel: 640 nm) at 60× water immersion mode using confocal laser scanning microscopy (LEICA TCS SP8).

Flow cytometry (FCM) assay

HepG2 and A373 cells were seeded onto a 24-well plate at a density of 1×10^5 cells per well. The cells were then cultured at 37 °C in a humidified atmosphere containing 5% CO_2 for a period of 24 h. Then preset concentration of GalNAc-Apc001-FAM, Apc001-FAM, GalNAc-Scramble, GalNAc-Apc001/Sclerostin-FAM mixture (1:1), Apc001/Sclerostin-FAM mixture (1:1), or GalNAc-Scramble/Sclerostin-FAM mixture (1:1) was added in the medium and the cells were incubated for a preset time. Following incubation, the cells were gently washed by 1×PBS buffer for three times to remove extracellular sample residues and then trypsinized. The cells were filtered by BD Falcon Cell Strainer (70 μm) and transferred to a tube. The flow cytometry analysis was conducted utilizing BD Accuri™ C6 Plus.

For internalization pathways, HepG2 cells were seeded onto confocal plates at a density of 8×10^4 cells in 2 mL DMEM medium. The cells were then cultured at 37 °C in a humidified atmosphere containing 5% CO_2 for a period of 24 h. Then, the cells were respectively pre-treated with 1 mM Amiloride, 20 μM Chlorpromazine, 500 μM Genistern for 10 mimutes, respectively, followed by treated with 1 μM GalNAc-Apc001/Sclerostin-FAM mixture for 2 h. Next, the cells were gently washed by 1×PBS buffer for three times to remove extracellular

sample residues and then trypsinized. The cells were filtered by BD Falcon Cell Strainer (70 μm) and transferred to a tube. The flow cytometry analysis was conducted utilizing BD Accuri™ C6 Plus.

Cumulation of sclerostin in cells

HepG2 cells A373 cells were seeded onto a 24-well plate at a density of 1×10^5 cells per well. The cells were then cultured at 37 °C in a humidified atmosphere containing 5% CO_2 for a period of 24 h. Then the cells were incubated with 100 nM GalNAc-Apc001/Sclerostin-FAM (1:1) mixture, Apc001/Sclerostin-FAM (1:1) mixture, and GalNAc-Scramble/Sclerostin-FAM (1:1) mixture, respectively, for varied time as required (0 h, 0.5 h, 1 h, 2 h, 4 h, 8 h). At the time point of 8 hour, the culture medium was removed and washed with 1×PBS buffer for three times. The cells were lysed by RIPA buffer on ice for 30 min and the FAM fluorescence of cell lysate was read by EnVision 2104 Multilabel Plate Reader (Perkin Elmer).

Adsorption capacity to hydroxyapatite

First, a standard curve was generated by measuring the fluorescence intensity of Cy3-labeled GalNAc-Apc001 at concentrations of 0, 0.312, 0.625, 1.25, 2.5, 5, and 10 μM. Next, 20 mg of HA suspensions were incubated with 3 μM Cy3-labeled GalNAc-Apc001 under controlled conditions. The mixtures were gently agitated at room temperature for 5 hours to facilitate binding. After incubation, the samples were centrifuged at 5000 rpm to pellet the HA particles and their bound complexes. The fluorescence intensity of the supernatant, containing unbound GalNAc-Apc001, was measured using a spectrofluorometer at the appropriate excitation and emission wavelengths for Cy3. The amount of compound adsorbed was determined by the decrease in supernatant fluorescence intensity compared to the initial solution, using the standard curve to convert fluorescence units to concentration.

Western blot assay

HepG2 cells were seeded onto a 24-well plate at a density of 1×10^5 cells per well. The cells were then cultured at 37 °C in a humidified atmosphere containing 5% CO_2 for a period of 24 h. Then the cells were incubated with 500 nM GalNAc-Apc001/Sclerostin (1:1) mixture for varied time as required (0 h, 1 h, 2 h, 4 h, 8 h). At the time point of 8 h, the culture medium was exchanged to normal medium to visualize the degradation of sclerostin until to 18 h. The cells were washed with 1×PBS buffer for three times and lysed by RIPA buffer on ice for 30 min. The supernatant was collected, and the concentration of protein was approximately determined

using Nanodrop 2000 roughly. Electrophoresis of equal amounts of the cell lysate were performed by 12% SDS-PAGE gel, followed with PVDF membrane transfer and non-specific blocking by 5% skim milk in TBST buffer. Then the membrane was treated with primary antibody (1:1000 diluted in 5% skim milk) overnight at 4 °C. Wash the membrane with 1×TBST buffer for three times gently on a shaker and further treated with secondary antibody (1:5000 diluted in 5% skim milk) for 1 h at room temperature. After washed with TBST buffer for three times, the membrane was treated with BeyoECL Plus substrate for 2 min and image by ChemiDoc Imaging Systems (Bio-Rad).

Biostability assay.

All biostability assays were performed in DMEM that contained 1 μM of tested oligonucleotide with the presence of 0.5 mg/mL SD rat liver microsomes (LMs), 20% fetal bovine serum (FBS, v/v) or 20 mU/mL snake venom phosphodiesterase (SVPDE) at 37 °C. Multiple time points were collected for each condition by quenching 30 μL of the reaction solution using 10 μL of deionized formamide that contained 0.5M ethylenediaminetetraacetic acid (EDTA). Samples were denatured for 10 min at 95 °C and analyzed by 20% denaturing polyacrylamide gel electrophoresis (PAGE). Gels were visualized using ChemiDoc Imaging Systems (Bio-Rad).

Wnt-signaling activation assay

HEK293 cells were cultured at 37 °C under humidified atmosphere and 5% CO_2 with Dulbecco's modified Eagle's medium (DMEM) containing 10% Fetal Bovine Serum (FBS) and 1% Penicillin-Streptomycin (PS). The cells were seeded on 24-well plate at a density of 1×10^5 cells in 0.5 mL of DMEM medium.After 24 hours, a Top flash plasmid (Firefly luciferase, 100 ng/well), a SV40 plasmid (Renilla luciferase, 10 ng/well), and a Wnt-1 plasmid (200 ng/well) were co-transfected into the cells using lipofectamine 3000 (Lipofectamine 3000 1 μL/well, P3000 reagent 1 μL/well). During the process, the plasmids and lipofectamine 3000 were first mixed in 25 μL OptiMem medium as buffer A. The P3000 reagent was dissolved in 25 μL OptiMem mediumas buffer B. Buffer A and buffer B were mixed and incubated for 15 min. Then, the mixture was directely added into culture medium and incubated for 6 h. Subsequently, the transfection medium was removed and 1 mL fresh DMEM medium containing 100 nM sclerostin was added. Then 2×10^5 co-culture cells prepared in advance (either HepG2, A375 or none) were added in a transwell and co-incubated, followed with the addition of 500 nM samples that needed to be varified (either GalNAc-Apc001, Apc001, GalNAc-Scramble or Scl-Antibody). After incubating at 37 °C for 12 h, the medium was removed and the cells were

lysed in 200 μL 1× passive lysis buffer (PLB) by shaking the culture vessel for 15 min at room temperature. Then, 15 μL lysate was transferred to 96-well OptiPlate to determine the luciferase activity by measuring the chemiluminescence using MD SpectraMax i3X Multi-Mode Microplate Reader system under the manufacturing protocol of Dual-Luciferase Reporter Assay System.

Real-time qPCR

MC3T3-E1 (mouse preosteoblast, ATCC CRL-2593) cell line was cultured in MEM-α medium supplemented with 10% FBS and 100 units/mL penicillin-streptomycin. After seeded in 24 well plates with the density of 1×10^5 cells/well, the cells were cultured for 24 h. Wnt-1 plasmid (200 ng/well) were co-transfected into the cells using lipofectamine 3000 (Lipofectamine 3000 1 μL/well, P3000 reagent 1 μL/well). During the process, the plasmids and lipofectamine 3000 were first mixed in 25 μL MEM-α medium (Omacgene) as buffer A. The P3000 reagent was dissolved in 25 μL OptiMem mediumas buffer B. Buffer A and buffer B were mixed and incubated for 15 min. Then, the mixture was directely added into culture medium and incubated for 6 h. Subsequently, the transfection medium was removed and 1 mL fresh DMEM medium containing 100 nM sclerostin was added. Then 2×10^5 another cells prepared in advance (either HepG2, A375 or none) were added in a transwell and co-incubated, followed with the addition of 500 nM samples that needed to be varified (either GalNAc-Apc001, Apc001, GalNAc-Scramble or Scl-Antibody). After incubating at 37 °C for 12 h, the medium was removed, and total RNAs were isolated by TransZol Up Plus RNA Kit according to the manufacturer's instructions. After purification, the total RNA was quantified by Nanodrop 2000 (ThermoFisher) and reversely transcribed into cDNA. The mRNA levels of bone formation markers were determined utilizing TaqMan Gene Expression Assays. Corresponding primers used in this assay were purchased from Biosyntech, including *Runx2* (Forward primer: CCTGAACTCTGCACCAAGTCCT, Reverse primer: TCATCTGGCTCAGATAGGAGGG), *Alp* (Forward primer: CCAGAAAGACACCTTGACTGTGG, Reverse primer: TCTTGTCCGTGTCGCTCACCAT), and *Ocn* (Forward primer: GCAATAAGGTAGTGAACAGACTCC, Reverse primer: CCATAGATGCGTTTGTAGGCGG), *Gapdh* (Forward primer: CATCACTGCCACCCAGAAGACTG, Reverse primer: ATGCCAGTGAGCTTCCCGTTCAG). Quantitative PCR reactions were conducted on the 7900 HT Sequence Detection System (Applied Biosystems) using the TaqMan Universal PCR

Master Mix. The relative mRNA expression was determined by the $2^{-\Delta\Delta Ct}$ method using ViiA 7 Real-Time PCR System (Thermo Lifetech).

Biodistribution *in vivo*

The Laboratory Animal House of Hong Kong Baptist University provided housing for the mice used in this study. The animal facility maintained a regulated environment with controlled temperature and a 12 h light/dark cycle, while food and water were freely accessible to the mice throughout the study. Prior to conducting any experiments, a minimum of one week was allocated for the mice to acclimate to their new environment. All *in vivo* studies were conducted in compliance with ethical guidelines and received approval from the Animal Experimentation Ethics Committee of the Hong Kong Baptist University (REC/22-23/0121).

For the biodistribution of GalNAc-Apc001, eight twelve-week-old C57BL/6 mice were randomly divided into four groups (two mice in each group) for further experimentation. The mice were administered with Cy3-labeled Apc001, Cy3-labeled GalNAc-Apc001 and Cy3-labeled scApc001 (scrambled aptamer) via subcutaneous injection at a dosage of 10 nmol, and the vehicle group was administered with equivolume of PBS. After 2 h of treatment, the mice were anesthetized using 2.5% Avertin, euthanized and vital organs were collected (heart, liver, spleen, lung, kidney and bone). The Cy3 fluorescence intensity in these organs was detected in the channel at Ex/Em: 520/570 nm using IVIS® Lumina X5 (PerkinElmer).

For the biodistribution of sclerostin delivered by GalNAc-Apc001, eight twelve-week-old C57BL/6 mice were randomly divided into four groups (two mice in each group) for further experimentation. The mice were pre-treated with Cy3-labeled sclerostin at a dosage of 1 nmol via intraperitoneal injection, followed by administration of Apc001 or GalNAc-Apc001 via subcutaneous injection at a dosage of 10 nmol. The vehicle group was administered with equivolume of PBS. After 2 h of treatment, the mice were anesthetized using 2.5% Avertin, euthanized and vital organs were collected (heart, liver, spleen, lung, kidney). The Cy3 fluorescence intensity in these organs was detected in the channel at Ex/Em: 520/570 nm using IVIS® Lumina X5 (PerkinElmer).

Biochemical analysis

Sixteen C57BL/6 mice were randomly divided into four groups (four mice in each group) for further experimentation. The mice were administered with Vehicle, Apc001, GalNAc-Apc001 and Scl-Antibody once a week for four weeks via subcutaneous injection at a dosage of 25 $mg\cdot kg^{-1}$, and the vehicle group was administered with equivolume of PBS. At the end of the

treatment, the mice were anesthetized using 2.5% Avertin, and the blood samples were collected in lithium heparin tubes. Next, the lithium heparin anticoagulated blood samples were centrifuged for 15 min in 2000 rcf at 4°C. Plasmas were analyzed with the cobas c 503 (Roche). The samples with small volumes were subjected to 1:1 or 1:3 manual dilution with 0.9% sodium chloride (B. Braun) before analysis. The parameters of albumin (ALB), alkaline phosphatase (ALP), alanine aminotransferase (ALT), aspartate aminotransferase (AST), total protein (TP), urea (UREA) and serum creatinine (CREJ) were measured.

Enzyme-Linked Immunosorbent Assay (ELISA) analysis

A monoclonal antibody specific to mouse SOST was pre-coated onto a microplate. Standards, prepared as serial dilutions at concentrations of 400, 200, 100, 50, 25, 12.5, and 6.25 pg/mL, and diluted (1:4) serum samples from the Vehicle, Apc001, and GalNAc-Apc001 groups were added to the wells. After incubation and washing to remove unbound substances, a biotin-labeled detection antibody was added. Following another wash, streptavidin-Horseradish Peroxidase (HRP) conjugate was introduced. After a final wash step, a TMB substrate solution was added for color development, which was stopped with sulfuric acid. The optical density (OD) was measured at 450 nm, and the SOST concentration in unknown samples was determined by interpolating their OD values from the standard curve generated using the serial dilutions.

Bone histomorphometry analysis

Prior to euthanasia, all animals were intraperitoneally injected with 50 μL of calcein (20 $mg \cdot kg^{-1}$) on the 13th and 3rd days. After anesthesia and sacrifice, the left femurs and left tibias were collected as bone tissue samples. These samples were fixed in 4% paraformaldehyde for 48 h, and dehydrated in increasing concentrations of sucrose (10%, 20%, and 30% in 1×PBS) for 24 h at each concentration, and then embedded in an optimal cutting temperature compound (Sakura Finetek, Co. Ltd., Tokyo, Japan) without decalcification. Longitudinal sections were obtained from the distal femurs and proximal tibias, and histomorphometric analyses of trabecular bone were conducted. The frozen tissue specimens, with a thickness of 5 μm, were obtained using a CryoStar NX50 (Thermo Fisher Scientific, Waltham, MA, USA). Fluorescence micrographs of the calcein-labeled bone sections were captured using a Q500MC fluorescence microscope (Leica, Bensheim, Germany). The parameters for bone dynamic histomorphometric analysis of trabecular bone, including bone formation rate (BFR/BS), were determined using a professional histomorphometric analysis system (BIOQUANT OSTEO,

Nashville, TN, USA). The calculated parameters were presented according to the standardized nomenclature (set by The American Society for Bone and Mineral Research).

Bone mechanical test

After anesthetizing and sacrificing the animals, the right femora and the fifth lumbar vertebrae (Lv5) and were immediately stored at -80 °C. The compression test and three-point test were performed using a universal testing machine (H25KS Series, Hounsfield Test Equipment Ltd, Redhill, UK) equipped with a 2.5 kN load cell. For the three-point experiment, the femora were loaded in the anterior-posterior direction with a span of 17 mm. The load was applied at the mid-shaft of the femur using a constant displacement rate of 1 $mm \cdot min^{-1}$. After failure, the load vs. displacement curves were recorded. The failure force (N) were calculated for statistical analysis. For the compression experiment, the fifth lumbar vertebrae were isolated from the vertebral columns and shaped into cylinders with two parallel planes measuring 5-7 cm. The cylinders were then positioned horizontally on the base. A constant displacement rate of 1 $mm \cdot min^{-1}$ was applied, and the load vs. displacement curves were recorded. The failure force (N) were calculated for statistical analysis.

Micro-CT analysis

The cortical microarchitecture of the left femoral mid-shaft, as well as the bone mass and trabecular microarchitecture analysis of the trabecular bone at the left proximal tibia metaphysis and the left distal femoral metaphysis, were assessed using micro-CT (version 6.5, vivaCT40, SCANCO Medical AG, Bassersdorf, Switzerland). The femur and tibia images were reconstructed and calibrated with an isotropic voxel size of 12.5 μm and 17.5 μm, respectively (70 kVp, 114 μA, 200 ms integration time, 260 thresholds, 1200 mg HA/cm3). Consistent filtering and segmentation values were applied to all measurements. The Scanco evaluation software was used to define regions of interest (ROIs) for trabecular parameters. For the trabecular bone analysis, a central region equivalent to 70% of the vertebral body height was selected, extending from the proximal end of the distal growth plate towards the vertebral body. In mice, 100 sequential slices were chosen for analysis, starting from 0.1 mm beyond the point where both condyles of the proximal tibia and distal femur were no longer visible. To ensure the ROI was within the endosteal envelope, we manually drew the trabeculae ROI on each of the 100 sequential slices. Trabecular bone parameters such as trabecular connectivity density (Tb. conn.D), trabecular volumetric mineral density (Tb. vBMD), and trabecular bone volume fraction (Tb. BV/TV) were calculated to evaluate trabecular bone mass. Additionally, trabecular

number (Tb. N), trabecular thickness (Tb. Th), and trabecular separation (Tb. Sp) were determined. For the proximal tibia and distal femur, 100 sequential slices were measured at the exact center and at the distal 50% of the femur length using an automated thresholding algorithm. Trabeculae in contact with cortical bone were manually excluded from the ROI for accurate analysis.

Histological examination

The paraffin sections underwent a sequence of immersion procedures, starting with treatment using Environmentally Friendly Dewaxing Transparent Liquid I and II, Anhydrous ethanol I and II, and 75% Ethyl alcohol, followed by rinsing with tap water. On the other hand, the frozen sections were thawed, fixed with a tissue fixative solution, and then rinsed with running water. For Hematoxylin staining, the sections were immersed in Hematoxylin solution, followed by the application of Hematoxylin Differentiation solution and Hematoxylin Bluing solution, with rinsing steps in between. Eosin staining was carried out by sequentially placing the sections in 85% ethanol, 95% ethanol, and Eosin dye. Dehydration and sealing processes involved the use of various ethanol and xylene solutions, concluding with sealing using neutral gum. Subsequently, microscope inspection, image acquisition, and analysis were performed, revealing blue nuclei and red cytoplasm upon result interpretation.

Immunohistochemistry

The removal of wax from the liver tissue sections was done by soaking the sections in a set of eco-friendly solutions and then in different strengths of pure ethanol. Afterward, the sections were washed with distilled water. Next, to make the antigens in the tissues accessible, we used a special buffer. We then gently shook the slides in a saline solution (PBS). Then, we treated the sections with a solution of 3% hydrogen peroxide. We washed them with PBS again. Next, we covered the sections with a 3% BSA solution to block unwanted antibody reactions. We added primary antibodies, specifically IL-6 and TNF-α, followed by a secondary antibody, washing with PBS after each antibody application. After that, we applied a DAB stain and then used hematoxylin to outline the cell structures. We dried the sections with alcohol and cleared them with xylene. Finally, we examined the slides under a microscope. This careful process made sure we removed all the wax, uncovered the antigens, stopped unwanted peroxidase actions, prevented false antibody reactions, and stained the tissues effectively. With this approach, we aimed to accurately detect and study the specific antigens in the tissues.

Statistical analysis

The data were presented as mean ± standard deviation for all variables. To assess inter-group differences in the study variables, including *in vitro* Wnt-induced signaling, *in vitro* mRNA levels of bone formation biomarkers, *in vivo* micro-CT parameters, *in vivo* bone histomorphometric parameters, and *in vivo* mechanical tests, a one-way ANOVA with Tukey's post-hoc test was performed. For distribution studies, statistical significance was determined using unpaired t-test. The statistical analysis was conducted using Origin 2019b and GraphPad Prism software, with a significance level set at $P < 0.05$. For *in vivo* study, the animals were randomly and blindly grouped by researchers. Animals in poor body condition were excluded from the analysis to ensure data integrity.

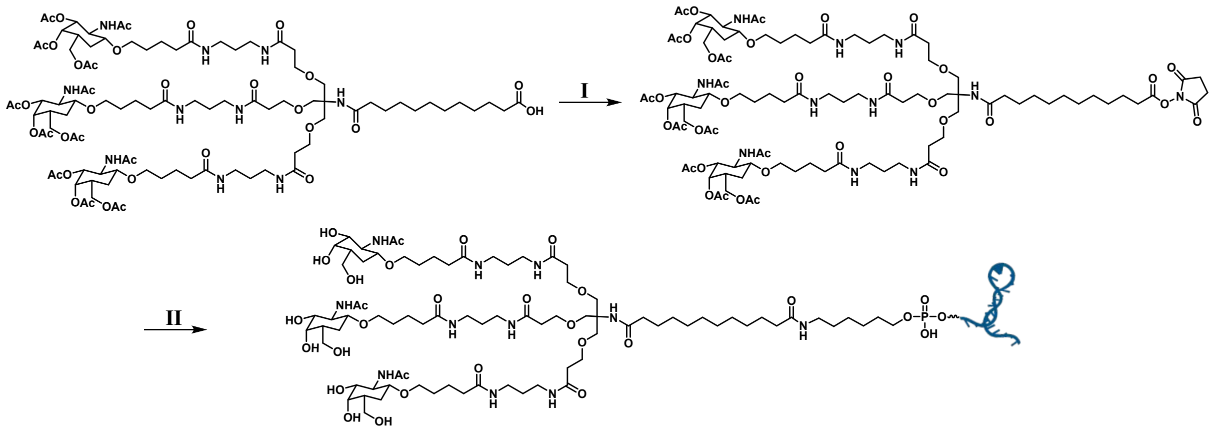

Scheme S1. The synthetic route of GalNAc-Apc001. Reagent and Condition: (i) N-hydroxysuccinimide (NHS), 1-ethyl-3-(3-dimethylaminopropyl)carbodiimide (EDCI), dichloromethane (DCM); (ii) Apc001, 1.0 M triethylamine acetate (TEAA), ammonium hydroxide.

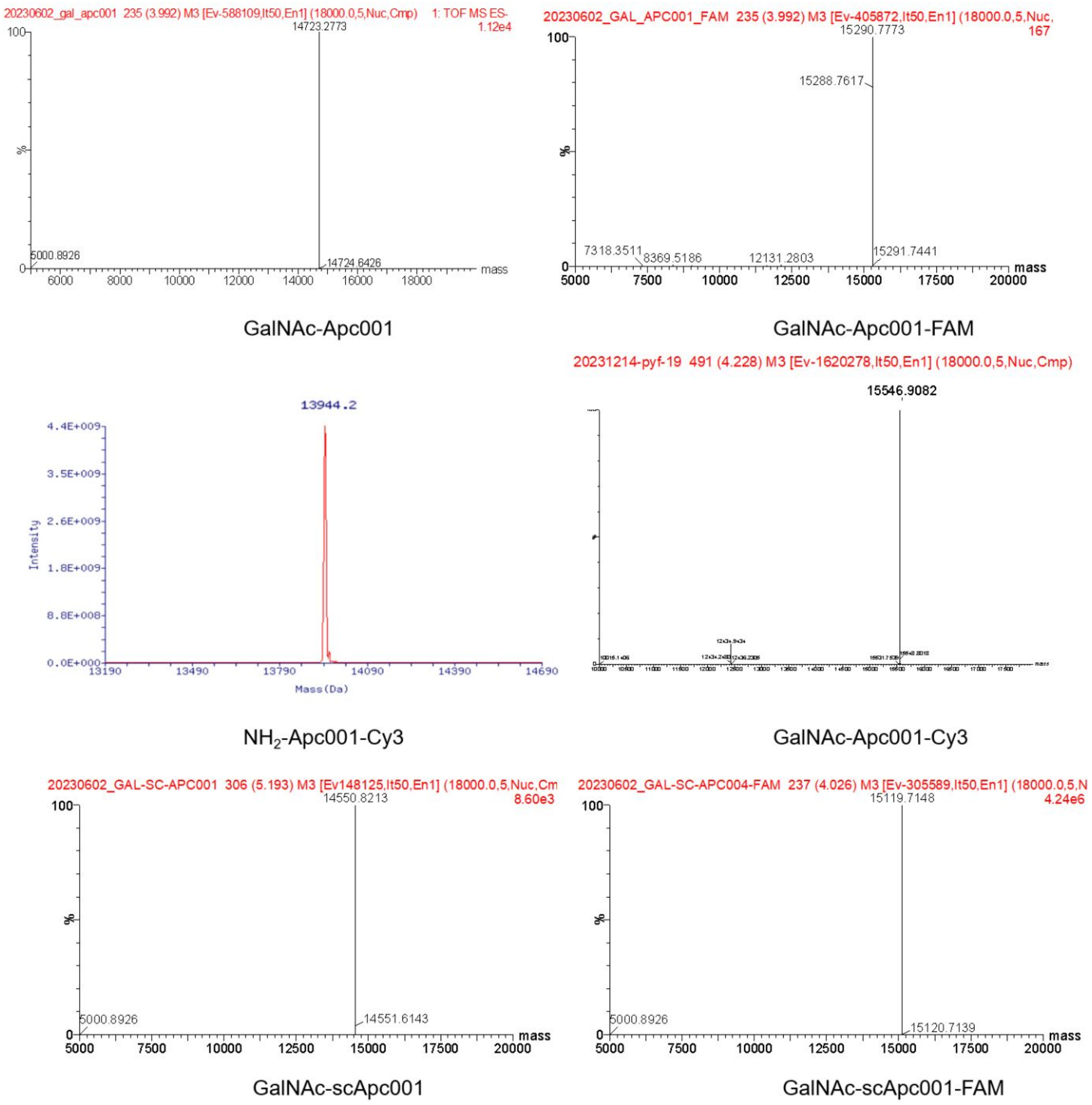

Fig. S1. ESI MS analyses of GalNAc-DNAs.

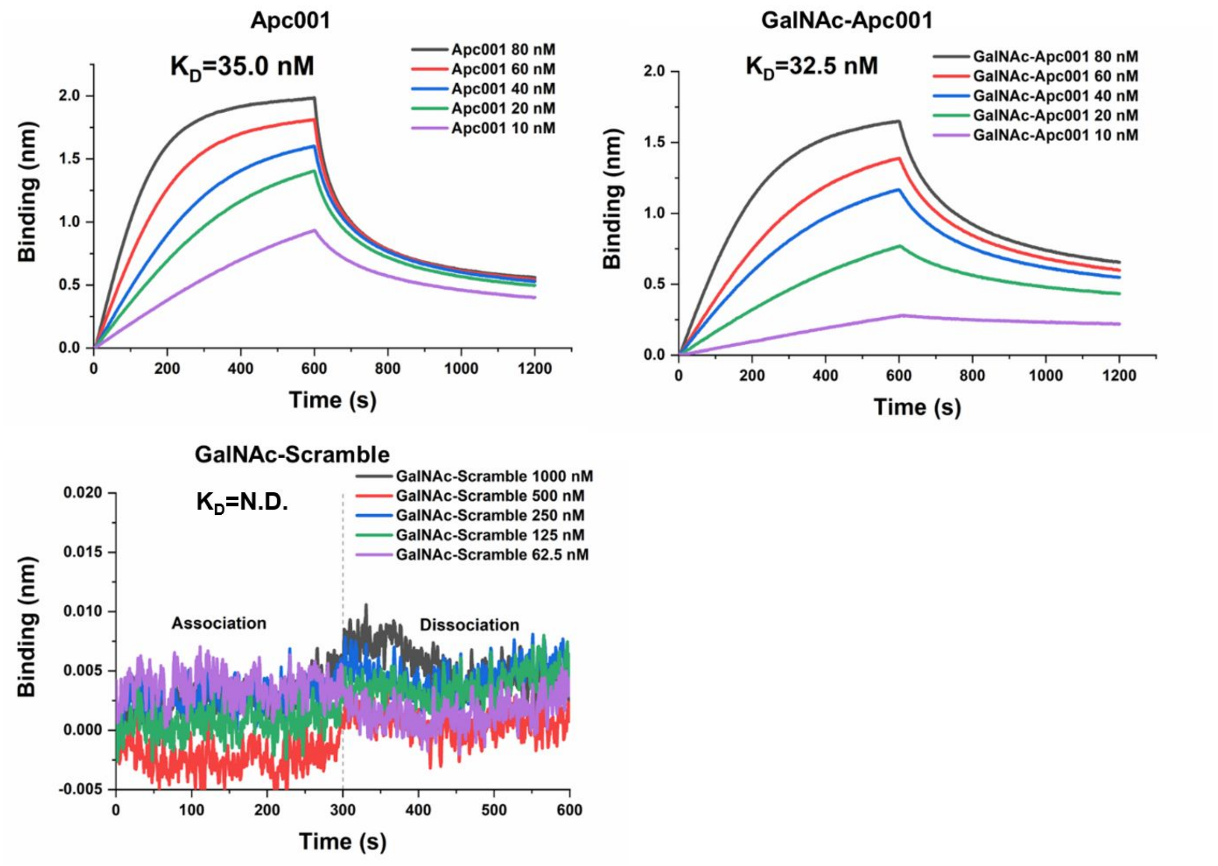

Fig. S2. The affinity measurement results of Apc001, GalNAc-Apc001, GalNAc-Scramble against sclerostin, detected by Biolayer interferometry. Note: N.D. represented Not detected.

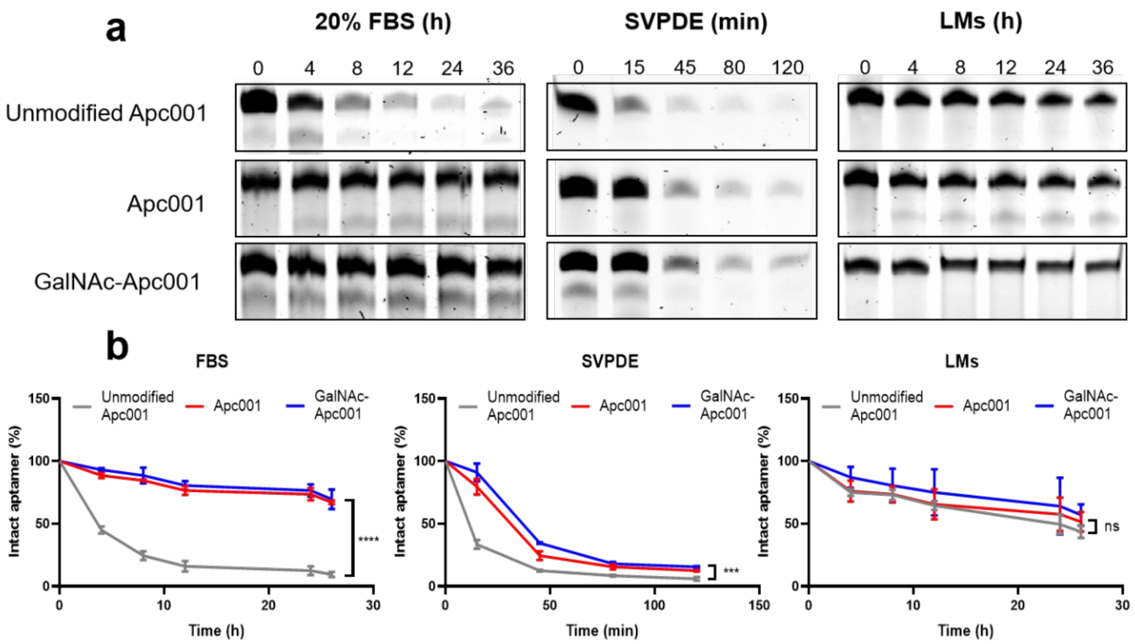

Fig. S3. Biostability of aptamers with and without GalNAc conjugation at each terminus. (**a**) Representative images of unmodified Apc001, Apc001, and GalNAc-Apc001 (1 µM per group) following incubation with fetal bovine serum (FBS, 20% v/v), snake venom phosphodiesterase

(SVPDE, 20 mU/mL), and liver microsomes (LMs, 0.5 mg/mL). (**b**) Quantitative analysis of intact aptamers over time. The initial concentration was normalized to 100%. Data are expressed as mean ± standard deviation. Statistical significance was assessed using two-way ANOVA: ns (not significant), P > 0.05; ***P < 0.001; ****P < 0.0001.

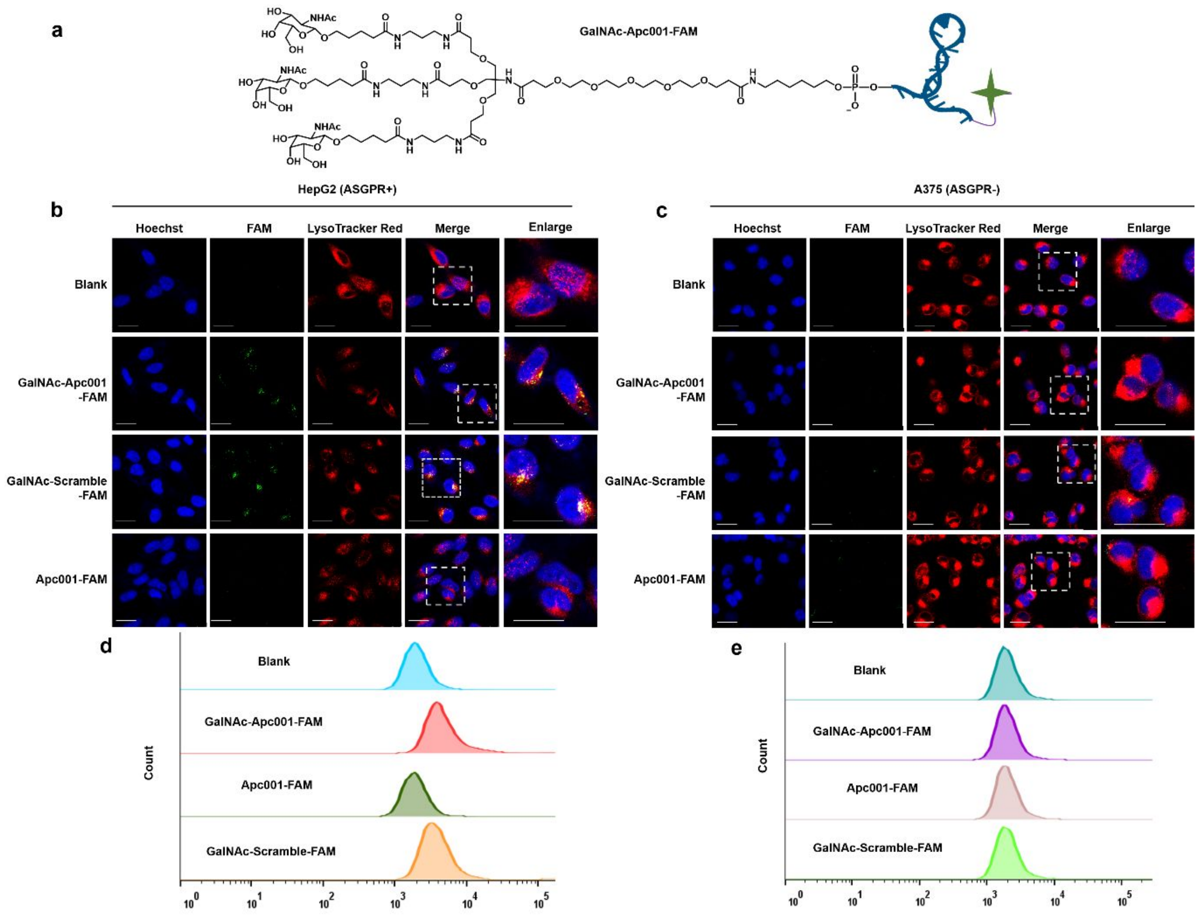

Fig. S4. GalNAc mediates cell-specific internalization and transport FAM-labeled Apc001 and Scramble sequence to ASGPR+ cell lysosomes. **b-c** Confocal microscopy images of HepG2 cells (**b**) and A375 cells (**c**) incubated with 1 μM GalNAc-Apc001-FAM, GalNAc-Scramble-FAM, and Apc001-FAM for 2 hours, respectively. Nuclei stained by Hoechst 33342 (Blue); FAM (Green); Lysosome stained by Lysotracker Red. Scale bar, 10 μm. **d-e** Flow cytometry analysis of the internalization of GalNAc-Apc001-FAM, GalNAc-Scramble-FAM, and Apc001-FAM, respectively, into HepG2 cells (**d**) and A375 (**e**). Sample concentration, 1 μM; Incubation time, 2 h

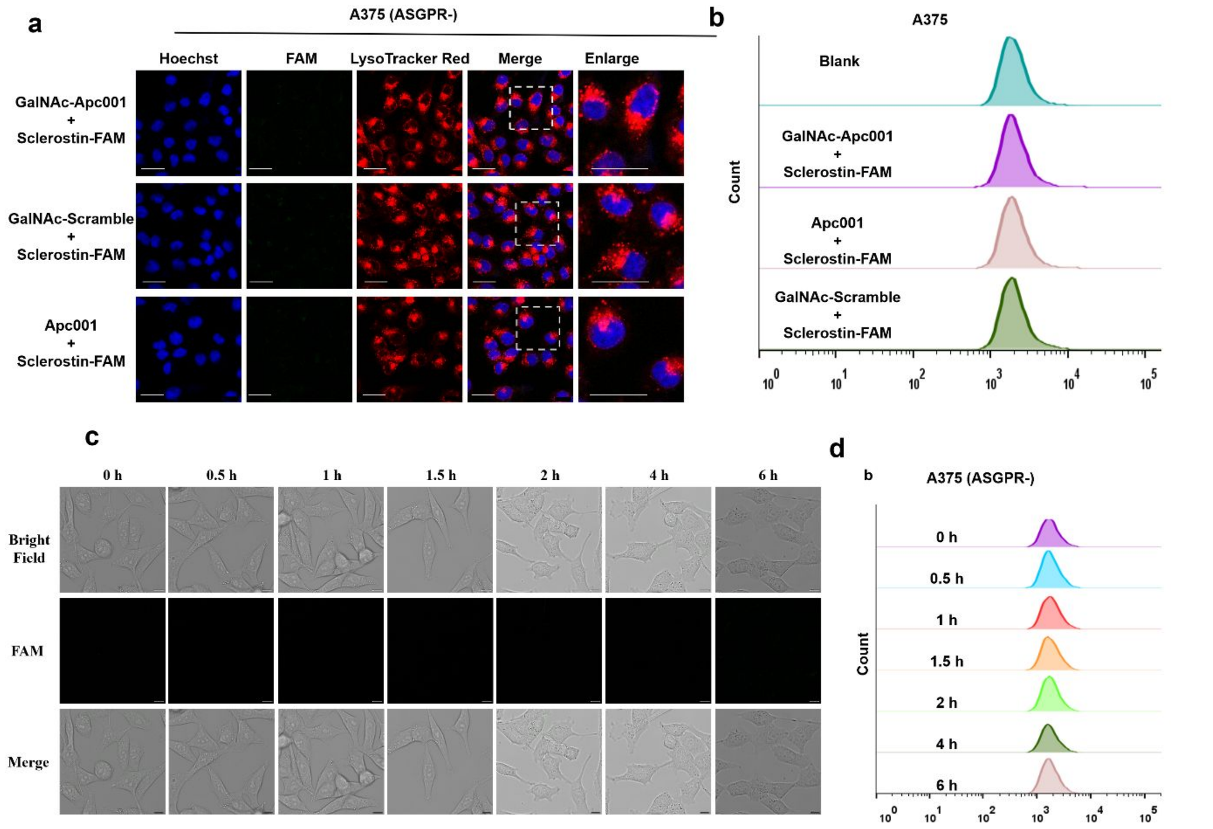

Fig. S5. Internalization of sclerostin mediated by GalNAc-Apc001 in ASGPR-negative A375 cells. (**a**) Confocal microscopy images of A375 cells incubated with 1 μM GalNAc-Apc001/Sclerositn-FAM, GalNAc-Scramble/Sclerositn-FAM, and Apc001/Sclerositn-FAM for 2 h, respectively. The samples were premixed at a 1:1 ratio. Nuclei stained by Hoechst 33342 (Blue); FAM (Green); Lysosome stained by Lysotracker Red. Scale bar, 25 μm. (**b**) Flow cytometry analysis of the internalization of GalNAc-Apc001/Sclerositn-FAM, GalNAc-Scramble/Sclerositn-FAM, and Apc001/Sclerositn-FAM into A375 cells. The samples were premixed at a 1:1 ratio. Incubation concentration, 1 μM; Incubation time, 2 h. (**c**) Time-dependent confocal microscopy images of A375 cells. The cells were incubated with DMEM medium containing 1 μM GalNAc-Apc001/Sclerostin-FAM (1:1) mixture. Scale bar, 10 μm. (**d**) Time-dependent flow cytometry analysis of the cell-specific internalization of Sclerostin-FAM mediated by GalNAc-Apc001 (1:1, 1 μM) into A375 cells.

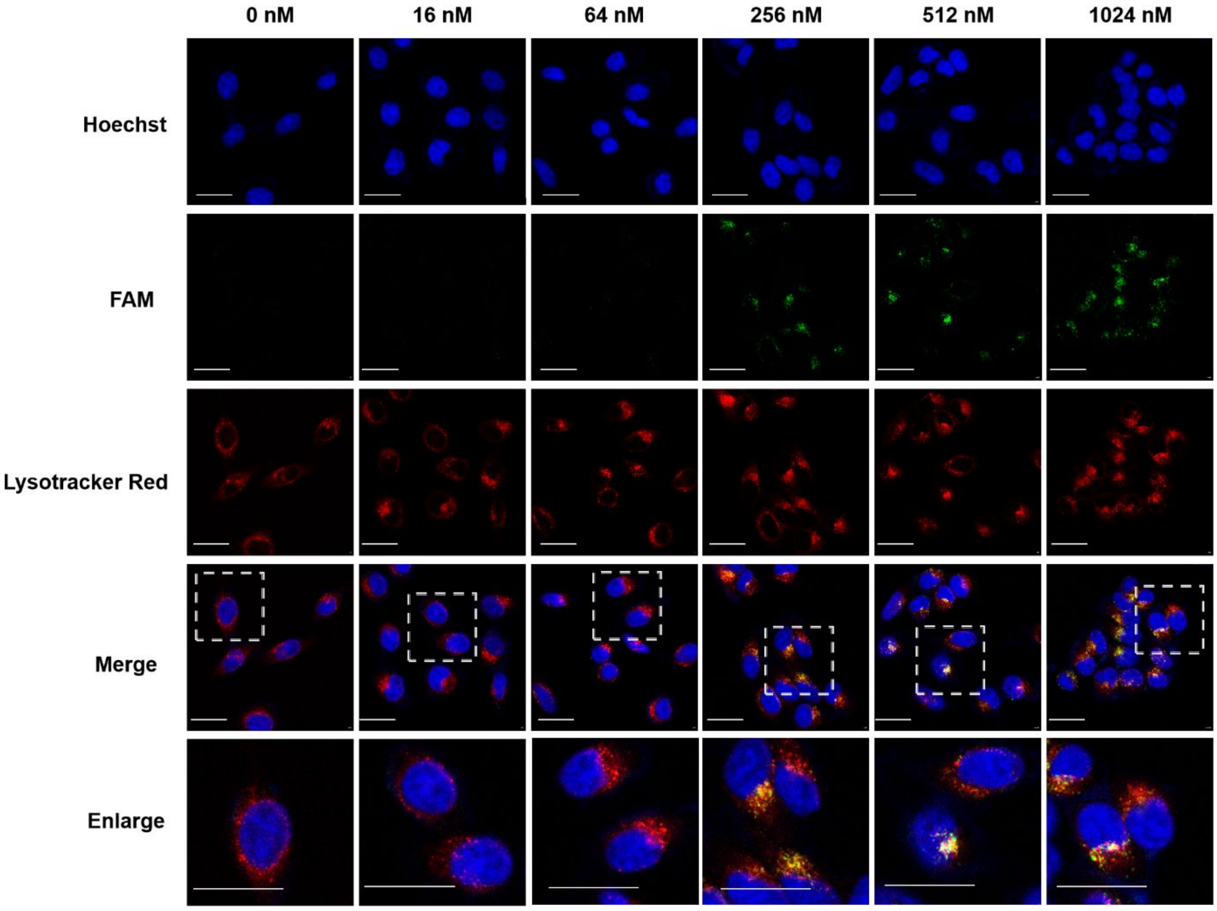

Fig. S6. The concentration-dependent cell internalization of sclerostin-FAM mediated by GalNAc-Apc001. HepG2 cells were incubated with DMEM medium containing varied concentration of GalNAc-Apc001/Sclerostin-FAM (1:1) mixture for 2 hours. Then the cells were imaged by confocal microscopy. Nuclei stained by Hoechst 33342 (Blue); FAM (Green); Lysosome stained by Lysotracker Red. Scale bar, 25 μm.

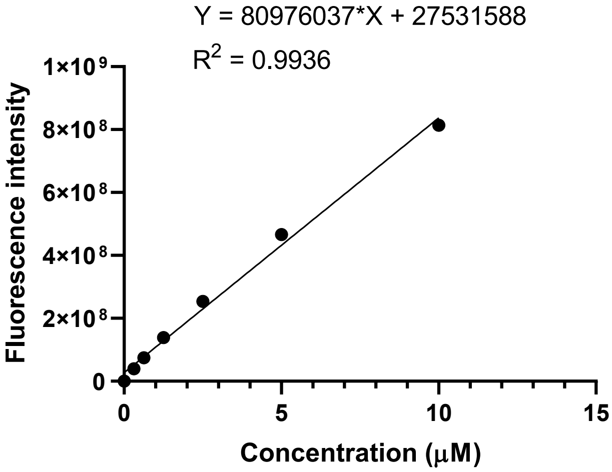

Fig. S7. Standard curve for Cy3-labeled GalNAc-Apc001 fluorescence. The fluorescence intensity was measured across a concentration range of 0 to 10 μM.

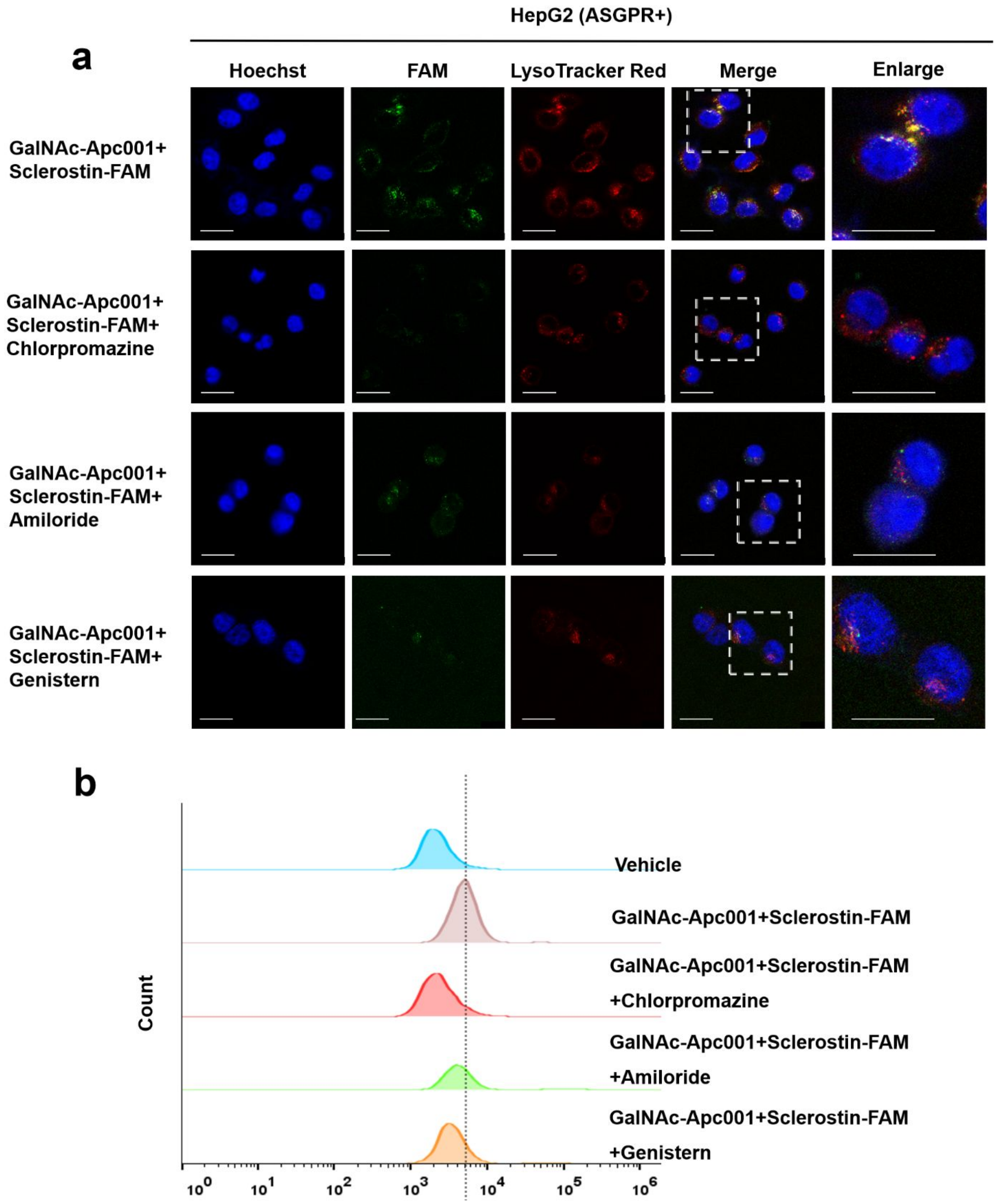

Fig. S8. Cellular internalization pathway of Sclerositn-FAM captured by GalNAc-Apc001. (**a**) Confocal microscopy images of HepG2 cells pre-treated with chlorpromazine, amiloride and genistein, followed by incubation with 1 μM GalNAc-Apc001/Sclerositn-FAM for 2 hours. Nuclei stained by Hoechst 33342 (Blue); FAM (Green); Lysosome stained by Lysotracker Red. Scale bar, 25 μm. The chlorpromazine, amiloride and genistein were used to suppress clathrin-mediated endocytosis, micropinocytosis and caveolae-mediated endocytosis, respectively. (**b**)

Flow cytometry analysis of the internalization of Sclerositn-FAM captured by GalNAc-Apc001. HepG2 cells were pre-treated with chlorpromazine, amiloride and genistein, followed by incubation with 1 μM GalNAc-Apc001/Sclerositn-FAM for 2 h.

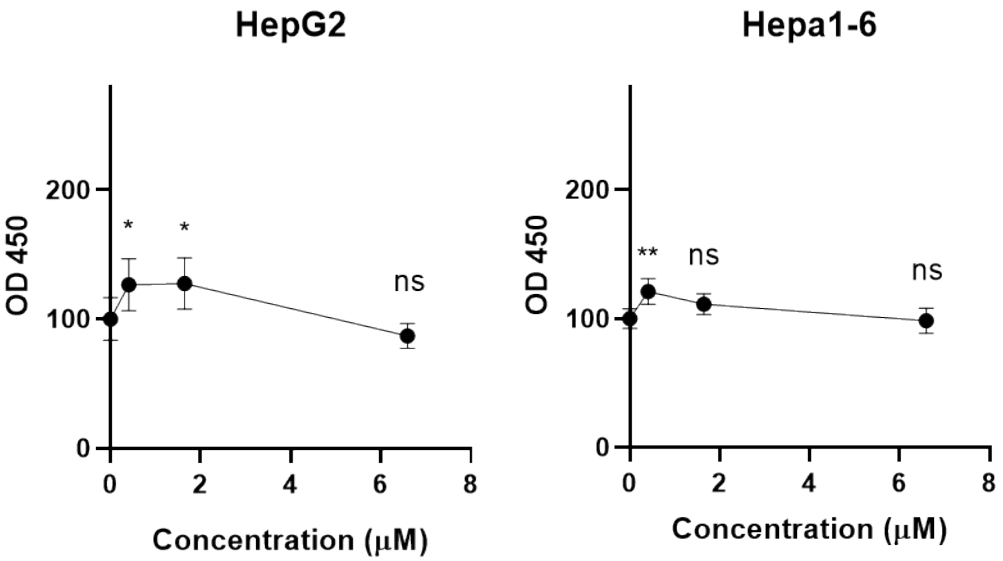

Fig. S9. The cytotoxicity of GalNAc-Apc001 on HepG2 cells and Hepa1-6 cells. Data were normalized by parameters in the PBS groups and expressed as mean ± standard deviation. One-way ANOVA with Tukey test was used to determine the inter-group differences, respectively. n = 6 per group, ns $P > 0.05$, * $P < 0.05$, ** $P < 0.01$.

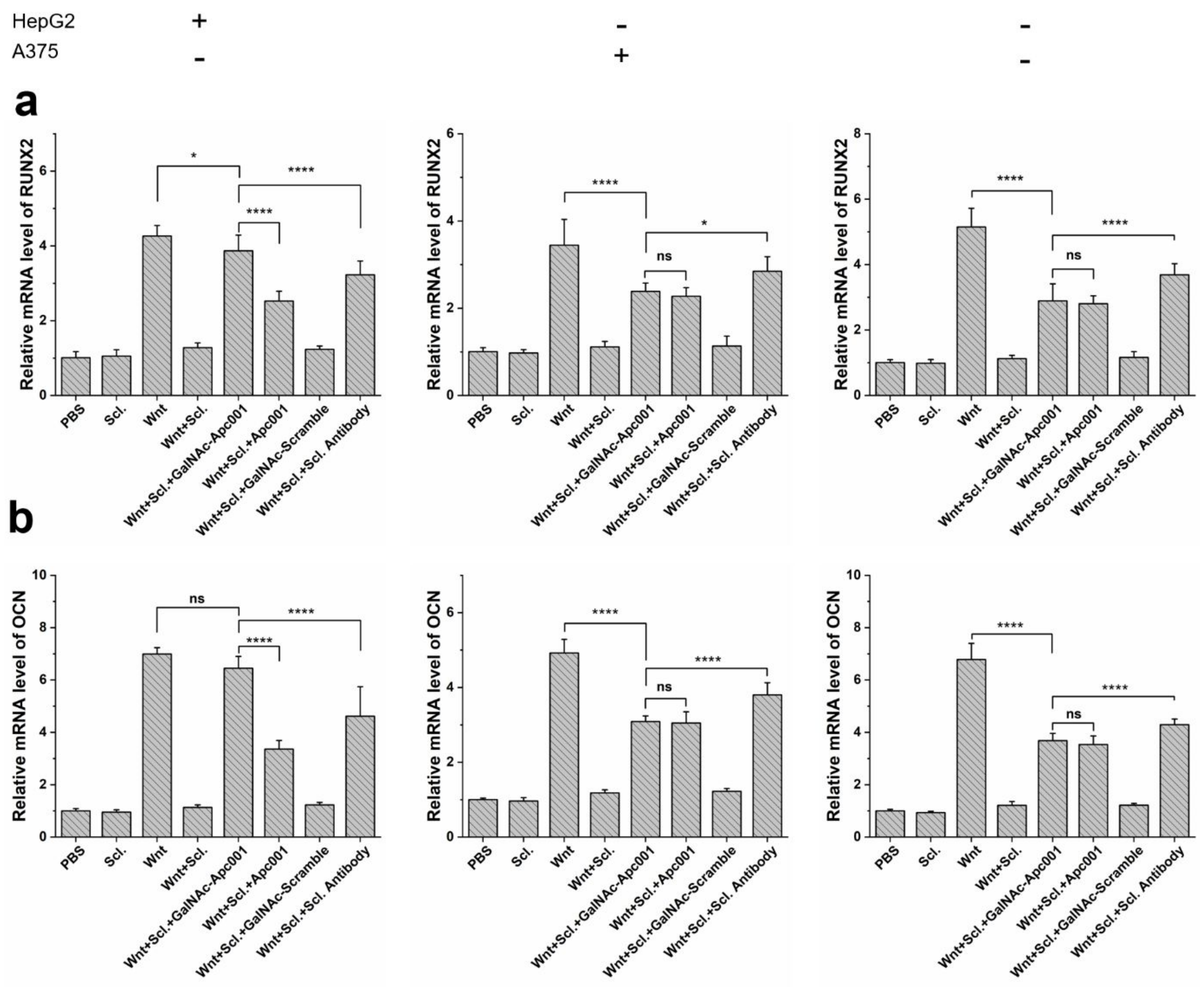

Fig. S10. The effect of GalNAc-Apc001, Apc001, GalNAc-Scramble and scl. Antibody on the down-regulated mRNA expression levels of bone formation markers, including RUNX2 (**a**) and OCN (**b**), induced by the sclerostin in MC3T3-E1 cells at the presence or absence of HepG2 cells. Data were normalized by parameters in the PBS groups and expressed as mean ± standard deviation. One-way ANOVA with Tukey test was used to determine the inter-group differences, respectively. n = 3 per group, * $P < 0.05$, ** $P < 0.01$, *** $P < 0.001$. Notes: PBS represented groups treated with 1× PBS which had the same volume with other groups. Scl. represented groups treated with 100 nM sclerostin. Wnt represented groups transfected with Wnt-1 plasmid. scl. Antibody represented groups treated with sclerostin antibody.

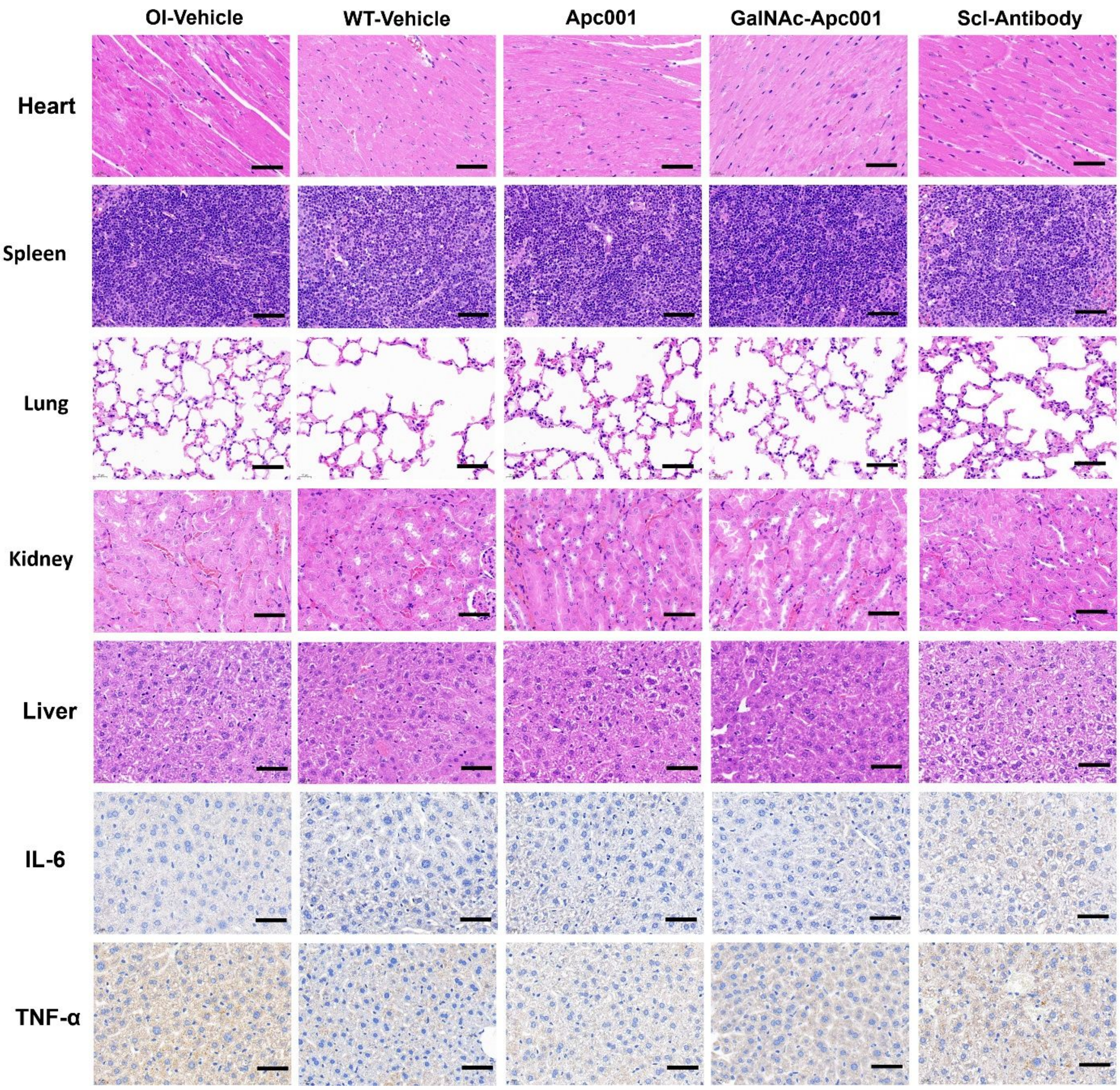

Fig. S11. Hematoxylin-eosin staining and immunohistochemical staining analysis of vital organs in mice after different treatment of Vehicle, Apc001, GalNAc-Apc001 and Scl-Antibody, respectively. The sections of heart, spleen, lung, kidney and liver from each group were stained by hematoxylin-eosin (H&E). IL-6 positive staining and TNF-α positive staining were indicated by brown, nuclei were stained by blue. n=3. Scale bar: 50 μm.

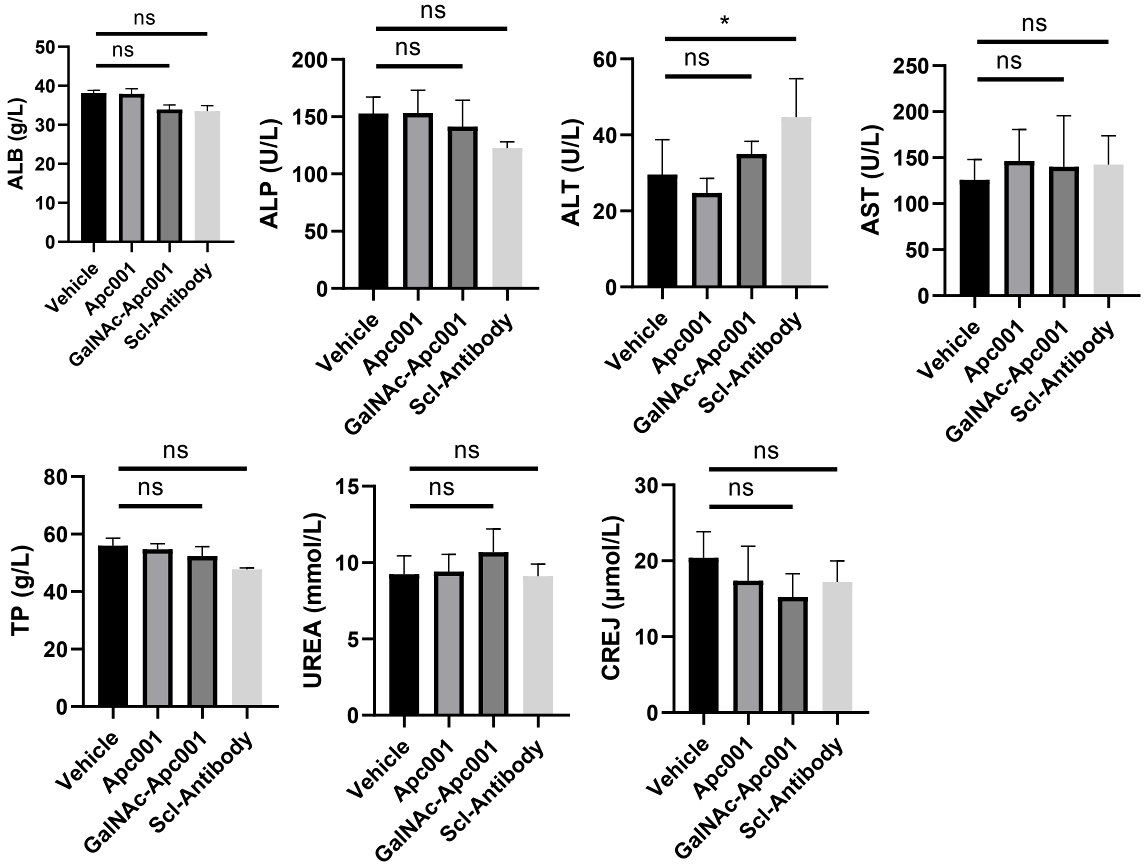

Fig. S12. The liver and kidney functions of the C57BL/6 mice treated with Vehicle, Apc001, GalNAc-Apc001 and scl-Antibody, respectively. Data were expressed as mean ± standard deviation followed by one-way ANOVA with Tukey's post-hoc test. n=4 per group. ns P > 0.05 versus Vehicle. Notes: ALB indicated albumin, ALP indicated alkaline phosphatase, ALT indicated alanine aminotransferase, AST indicated aspartate aminotransferase, TP indicated total protein, UREA indicated urea, CREJ indicated serum creatinine

Table S1. MS-identification of GalNAc-DNAs

Code	Name	Chemical Formula	Molecular Weight	Exact Mass [M]	Found [M]
1	GalNAc-Apc001	$C_{488}H_{663}N_{158}O_{295}P_{41}$	14732.4	14725.1	14723.3
2	GalNAc-Apc001-FAM	$C_{516}H_{689}N_{159}O_{305}P_{42}$	15299.9	15292.2	15290.8
3	NH2-Apc001-Cy3	$C_{453}H_{586}N_{151}O_{277}P_{42}S_2^-$	13943.5	13936.5	13944.2
4	GalNAc-Apc001-Cy3	$C_{526}H_{714}N_{161}O_{306}P_{42}S_2^-$	15553.3	15545.4	15546.1
5	GalNAc-scApc001	$C_{486}H_{664}N_{155}O_{288}P_{41}$	14555.4	14548.1	14550.8
6	GalNAc-scApc001-FAM	$C_{514}H_{690}N_{156}O_{298}P_{42}$	15122.9	15115.3	15119.7
